# Molecular Dissection of Pro-Fibrotic IL11 Signaling in Cardiac and Pulmonary Fibroblasts

**DOI:** 10.3389/fmolb.2021.740650

**Published:** 2021-09-28

**Authors:** Anissa A. Widjaja, Sivakumar Viswanathan, Dong Jinrui, Brijesh K. Singh, Jessie Tan, Joyce Goh Wei Ting, David Lamb, Shamini G. Shekeran, Benjamin L. George, Sebastian Schafer, David Carling, Eleonora Adami, Stuart A. Cook

**Affiliations:** ^1^ Cardiovascular and Metabolic Disorders Program, Duke-National University of Singapore Medical School, Singapore, Singapore; ^2^ National Heart Research Institute Singapore, National Heart Centre Singapore, Singapore, Singapore; ^3^ Boehringer Ingelheim, Immunology and Respiratory, Ingelheim am Rhein, Germany; ^4^ MRC-London Institute of Medical Sciences, London, United Kingdom

**Keywords:** interleukin-11, signaling, fibrosis, fibroblasts, nintedanib, IL11, IL11RA

## Abstract

In fibroblasts, TGFβ1 stimulates IL11 upregulation that leads to an autocrine loop of IL11-dependent pro-fibrotic protein translation. The signaling pathways downstream of IL11, which acts via IL6ST, are contentious with both STAT3 and ERK implicated. Here we dissect IL11 signaling in fibroblasts and study IL11-dependent protein synthesis pathways in the context of approved anti-fibrotic drug mechanisms of action. We show that IL11-induced ERK activation drives fibrogenesis and while STAT3 phosphorylation (pSTAT3) is also seen, this appears unrelated to fibroblast activation. Ironically, recombinant human IL11, which has been used extensively in mouse experiments to infer STAT3 activity downstream of IL11, increases pSTAT3 in *Il11ra1* null mouse fibroblasts. Unexpectedly, inhibition of STAT3 was found to induce severe proteotoxic ER stress, generalized fibroblast dysfunction and cell death. In contrast, inhibition of ERK prevented fibroblast activation in the absence of ER stress. IL11 stimulated an axis of ERK/mTOR/P70RSK protein translation and its selectivity for Collagen 1 synthesis was ascribed to an EPRS-regulated, ribosome stalling mechanism. Surprisingly, the anti-fibrotic drug nintedanib caused dose-dependent ER stress and lesser pSTAT3 expression. Pirfenidone had no effect on ER stress whereas anti-IL11 specifically inhibited the ERK/mTOR axis while reducing ER stress. These studies define the translation-specific signaling pathways downstream of IL11, intersect immune and metabolic signaling and reveal unappreciated effects of nintedanib.

## Introduction

TGFβ1 is one of the most studied human genes and has long been regarded as the dominant fibrogenic factor (Dolgin, 2017). While canonical TGFβ1-driven SMAD2/3 activation is central to its activity in fibroblasts, increased protein translation through non-canonical pathways is also important (Chaudhury et al., 2010; Schwarz, 2015; Zhang, 2017). TGFβ1-driven ERK activation has long been recognized as important and more recently STAT3 has been proposed as “*a key integrator of profibrotic signaling*” (Dees et al., 2012; Chakraborty et al., 2017; McHugh, 2017; Zhang, 2017). In 2017, we showed that autocrine IL11 activity is required downstream of TGFβ1-stimulated SMAD activation for fibroblast-to-myofibroblast transformation (Schafer et al., 2017). Intriguingly, the fibrogenic effects of IL11 across stromal cell types are evident only at the translational level (Widjaja et al., 2019; Cook and Schafer, 2020; Lim et al., 2020). To date, the pathways acting downstream of IL11, while seemingly ERK-dependent, remain largely unexplored.

IL11 is a little studied and rather misunderstood member of the IL6 family of proteins (Cook and Schafer, 2020; Widjaja et al., 2020). To signal, IL6 family cytokines bind to their cognate alpha receptors and the receptor:ligand complexes then bind to the common IL6ST (gp130) receptor. Canonical gp130 signaling involves its auto-phosphorylation, recruitment of Janus kinases, STAT3 activation and target gene transcription. It thus follows that STAT3 activity could underlie the pro-fibrotic effects of IL11/IL11RA/gp130 signaling in fibroblasts. However, this notion is incongruent with the fact that IL11 does not regulate STAT3 target gene transcription in stromal cells and accumulating data that suggest ERK as the dominant pathway (Widjaja et al., 2019; Cook and Schafer, 2020; Lim et al., 2020; Adami et al., 2021). We hypothesize that there was a pathway(s) downstream of IL11-stimulated ERK signaling that was of primary importance for pro-fibrotic protein translation.

Here, we set out to better understand how IL11 stimulates pro-fibrotic gene translation in fibroblasts by dissecting the relative contributions of ERK or STAT3 and studying downstream and related pathways. In these studies, we used neutralizing antibodies, fibroblasts from *Il11ra1* mice, and pharmacological approaches that identified IL11-activated the ERK/mTOR/P70S6K axis, which is of central importance for profibrotic gene translation. We discounted a role for STAT3 for fibroblast activation following TGFβ1 or IL11 stimulation and found instead that inhibition of STAT3 causes severe ER stress in activated fibroblasts. Informed by these data, we went on to examine the anti-fibrotic activities of pirfenidone and nintedanib in the context of the ERK/mTOR/P70S6K axis. While these drugs are approved for the treatment of fibrotic human diseases, their mechanisms of action (MOA) remain unclear and it is unknown if they affect IL11 signaling, which we addressed in concluding experiments (Schaefer et al., 2011; Roth et al., 2015).

## Materials and Methods

### Antibodies

BIP (3177, CST), Cleaved-Caspase 3 (9664, CST), Caspase 3 (9662, CST), CHOP (5554, CST), Collagen I (ab34710, Abcam), phospho-ERK1/2 (4370, CST), ERK1/2 (4695, CST), GAPDH (2118, CST), neutralizing anti-human gp130 (MAB628, R&D Systems), anti-gp130 (PA5-99526, Thermo Fisher), gp130 (PA5-28932, Thermo Fisher, IF), IgG (11E10, Aldevron), neutralizing anti-IL11 (X203, Aldevron), commercial anti-IL11 (MAB218, R&D Systems), neutralizing anti-IL11RA (X209, Aldevron), IL11RA (ab125015, abcam, IF), phospho-mTOR (2971, CST), mTOR (2972,CST), phospho-p70S6K (9205, CST), p70S6K (2708, CST), p-PDGFRB (3124, CST), PDGFRB (3169, CST), phospho-S6 ribosomal protein (4858, CST), S6 ribosomal protein (2217, CST), ⍺-SMA (ab7817, Abcam, Operetta), ⍺-SMA (19245, CST, WB), phospho-SMAD2 (3108, CST), SMAD2 (5339, CST), phospho-STAT3 (4113, CST), STAT3 (4904, CST), XBP-1 (sc-8015, SantaCruz), mouse Alexa Fluor 488 secondary antibody (ab150113, Abcam), mouse HRP (7076, CST), rabbit Alexa Fluor 488 secondary antibody (ab150077, Abcam), rabbit HRP (7074, CST).

### Recombinant Proteins

Commercial recombinant proteins: human TGFβ1 (PHP143B, Bio-Rad), human PDGF (220-BB-010, R&D Systems).

Custom recombinant proteins: Recombinant human IL11 (rhIL11, UniProtKB:P2o0809) and recombinant mouse IL11 (rmIL11, UniProtKB: P47873) were synthesized without the signal peptide. HyperIL-11 (IL11RA:IL-11 fusion protein), was constructed using a fragment of IL11RA (amino acid residues 1–317; UniProtKB: Q14626) and IL-11 (amino acid residues 22–199, UniProtKB: P20809) with a 20 amino acid linker (GPAGQSGGGGGSGGGSGGGSV) (Dams-Kozlowska et al., 2012). All custom recombinant proteins were synthesized by GenScript using a mammalian expression system.

### Chemicals

Cycloheximide (C1988, Sigma), 4′,6-diamidino-2-phenylindole (DAPI, D1306, Thermo Fisher), Halofuginone (sc-211579, SantaCruz), Nintedanib (S1010, Selleck Chemicals), Pirfenidone (P2116, Sigma), S3I-201 (SML0330, Sigma), Stattic (S7947, Sigma), U0126 (9903, CST), Thapsigargin (12758, CST), Tunicamycin (12819, CST).

### Cell Culture

Primary adult human cardiac fibroblasts (HCFs), primary adult mouse cardiac fibroblasts (MCFs), and primary adult human lung fibroblasts (HLFs) were grown and maintained at 37°C and 5% CO_2_. The growth medium was renewed every 2–3 days and cells were passaged at 80% confluence, using standard trypsinization techniques. All experiments were carried out at low cell passage (<P3). Cells were serum-starved overnight in basal media prior to stimulation. Cells were stimulated with different treatment conditions and durations, as outlined in the main text or figure legends. Stimulated cells were compared to unstimulated cells that have been grown for the same duration under the same conditions, but without the stimuli.

### Primary Human Cardiac Fibroblasts Culture

Primary HCFs (6330, Lot number 9580, ScienCell) were isolated from the healthy male heart. Cells were grown and maintained in FM-2 complete media which contains Fibroblast medium-2 (2331, ScienCell), Fibroblasts growth supplement-2 (FGS-2, 2382, ScienCell), 5% fetal bovine serum, and 1% Penicillin-streptomycin (P/S, 0353, ScienCell).

### Primary Mouse Cardiac Fibroblasts Culture

Primary MCFs were isolated from *Il11ra1-*deleted mice (*Il11ra* ^*−/−*^) and wild-type (WT, *Il11ra* ^*+/+*^) mice (B6.129S1-*Il11ra* ^*tm1Wehi*^/J, Jackson’s Laboratory). Atria were minced and digested with mild agitation for 30 min at 37°C in Dulbecco’s Modified Eagle Medium (DMEM, 11995-065, Gibco) containing 1% P/S and 0.14 Wunsch U ml^−1^ Liberase (5401119001, Roche). Fibroblasts were enriched via negative selection with magnetic beads against mouse CD45 (leukocytes), CD31 (endothelial) and CD326 (epithelial) using a QuadroMACS separator (Miltenyi Biotec) according to the manufacturer’s protocol. Fibroblasts were maintained and cultured in complete DMEM supplemented with 10% FBS and 1% P/S.

### Primary Human Lung Fibroblasts Culture

Primary HLFs (CC-2512, Lonza) were grown and maintained in FGM-2 complete media, which contains Fibroblast basal medium (CC-3131, Lonza) and hFGF-B, Insulin, fetal bovine serum, GA-1000 as growth supplements (FGM™-2 SingleQuots™, CC-4126, Lonza).

### Half Maximal Inhibitory Concentration Measurement

HCFs and MCFs were stimulated with IL11 (24 h) in the presence of IgG (11E10, 2 μg/ml; Aldevron) and varying concentrations of gp130 antibodies (MAB628 for HCFs and PA5-99526 for MCFs; 61 pg/ml to 4 μg/ml; 4-fold dilutions). Supernatants were collected and assayed for the amount of secreted MMP2. Dose-response curves were generated by plotting the logarithm of the gp130 antibodies tested concentrations (pM) versus the corresponding inhibition values (%), using the least squares ordinary fit. We considered MMP2 secretion by unstimulated cells as maximal inhibition (100%), while MMP2 secretion after stimulation with IL11 constituted 0% inhibition.

### siRNA Knockdown

HCFs were transfected using Lipofectamine RNAiMax (Life Technologies), following the manufacturer’s instructions for reverse transfection. Cells were transfected with 12.5 nM On-Targetplus siRNAs (siEPRS: L-008245-00-0005, siNT: D-001810-10-05, Dharmacon) in a medium consisting of serum-free Opti-MEM and complete FM-2 media in a 1:9 ratio. Following 24 h of transfection, cells were serum-starved overnight prior to stimulation with IL11, HyperIL11 or TGFβ1.

### Operetta High Throughput Phenotyping Assay

The Operetta assay was performed as previously described (Schafer et al., 2017) with minor modifications: HCFs were seeded in 96-well black CellCarrier plates (PerkinElmer) at a density of 5 × 10^3^ cells per well. Following simulations, cells were fixed in 4% PFA (Thermo Fisher), permeabilized with 0.1% Triton X-100 (Sigma) and non-specific sites were blocked with 0.5% BSA and 0.1% Tween-20 in PBS. Cells were incubated overnight (4°C) with primary antibodies (1:500), followed by incubation with the appropriate Alexa Fluor 488 secondary antibodies (1:1,000). Cells were counterstained with 1 μg/ml DAPI (D1306, Thermo Fisher) in blocking solution. Each condition was imaged from duplicated wells and a minimum of seven fields/well using Operetta high-content imaging system 1,483 (PerkinElmer). Cells expressing ACTA2 were quantified using Harmony v3.5.2 (PerkinElmer) and the percentage of activated fibroblasts/total cell number (⍺-SMA^+ve^) was determined for each field. The measurement of fluorescence intensity per area (normalized to the number of cells) of Collagen I was performed with Columbus 2.9 (PerkinElmer).

### Live/Dead Cells Quantification Assay

HCFs were seeded in 96-well black Cell Carrier plates at a density of 6 × 10^3^ cells/well. Following stimulation, cells were incubated 1 h with 1 μg/ml Hoechst 33342 (62249, Thermo Fisher Scientific) and DRAQ7 (D15106, Thermo Fisher Scientific; 1 μg/ml) in serum-free basal medium. Each condition was imaged from three wells and a minimum of 23 fields/well using Operetta high-content imaging system (1,483, PerkinElmer). Live and dead cells were quantified using Harmony v3.5.2 (PerkinElmer).

### Enzyme-Linked Immunosorbent Assay

The levels of IL11 and MMP2 in equal volumes of cell culture media were quantified using Human IL-11 Quantikine ELISA kit (D1100, R&D Systems) and Total MMP-2 Quantikine ELISA kit (MMP200, R&D Systems), respectively according to manufacturer’s instructions.

### Immunoblotting

For protein extraction and immunoblotting purposes, cells were seeded on 6-well plates at a density of 2 × 10^5^ cells. HCFs or MCFs were homogenized in RIPA Lysis and Extraction Buffer (89901, Thermo Scientific) containing protease and phosphatase inhibitors (Roche). Protein lysates were then separated by SDS-PAGE, transferred to PVDF membranes, blocked for 1 h with 3% BSA, and incubated overnight with the primary antibodies (1:1,000). Protein bands were visualized using the ECL detection system (Pierce) with the appropriate HRP (1:5000).

### Quantitative Polymerase Chain Reaction

Total RNA was extracted from HCF lysate using RNeasy column (Qiagen) purification and cDNAs were synthesized with iScript™ cDNA synthesis kit (Bio-Rad) according to manufacturer’s instructions. Gene expression analysis was performed with fast SYBR green (Qiagen) technology using StepOnePlus™ (Applied Biosystem) over 40 cycles. Expression data were normalized to *GAPDH* mRNA expression and fold change was calculated using 2^−∆∆Ct^ method. The primer sequences are as follow:

**Table:** 

Gene	Forward	Reverse
*EPRS*	5′-GTG​TTT​GGG​CCA​CCC​TAA​AAG-3′	5′-CTG​GAG​GAA​ATC​TGA​CGG​TAA​C-3′
*GAPDH*	5′-GGA​GTC​AAC​GGA​TTT​GGT​CG-3′	5′-ATC​GCC​CCA​CTT​GAT​TTT​GG3′

### OP-Puro Protein Synthesis Assay

HCFs were seeded in 96-well black CellCarrier plates (PerkinElmer) at a density of 5 × 10^3^ cells per well. Cycloheximide (100 μg/ml), a translation inhibitor used as a negative control, was added to the cells in basal medium, 1 hour prior to OPP labelling. OPP labelling was performed during the last treatment hour using Click-iT^®^ OPP reagent (C10456 kit, ThermoFisher Scientific) at a final concentration of 20 μM in basal medium. After incubation, cells were washed once with PBS and fixed using 4% paraformaldehyde in PBS for 15 min at room temperature (RT), and permeabilized using 0.5% Triton® X-100 for 15 min at RT. 100 μl Click-iT® OPP reaction cocktail containing Alexa Fluor™ 488 picolyl azide was added into each well and incubated for 30 min at RT, protected from light. Cells were washed with Click-iT® Reaction Rinse Buffer before proceeding for DNA staining, using HCS NuclearMask™ (1 μg/ml) Blue Stain following standard procedures. Images were acquired using the Operetta High Content Imaging system.

### Statistical Analyses

Statistical analyses were performed using GraphPad Prism software (version 8). Statistical significance between control and experimental groups were analysed by two-sided Student’s t tests or by one-way ANOVA as indicated in the figure legends. *p* values were corrected for multiple testing according to Dunnett’s (when several experimental groups were compared to a single control group) or Tukey (when several conditions were compared to each other within one experiment). The criterion for statistical significance was *p* < 0.05.

## Results

### Fibrogenesis is Associated With IL11-Induced ERK but not STAT3 Activation

**FIGURE 1:**
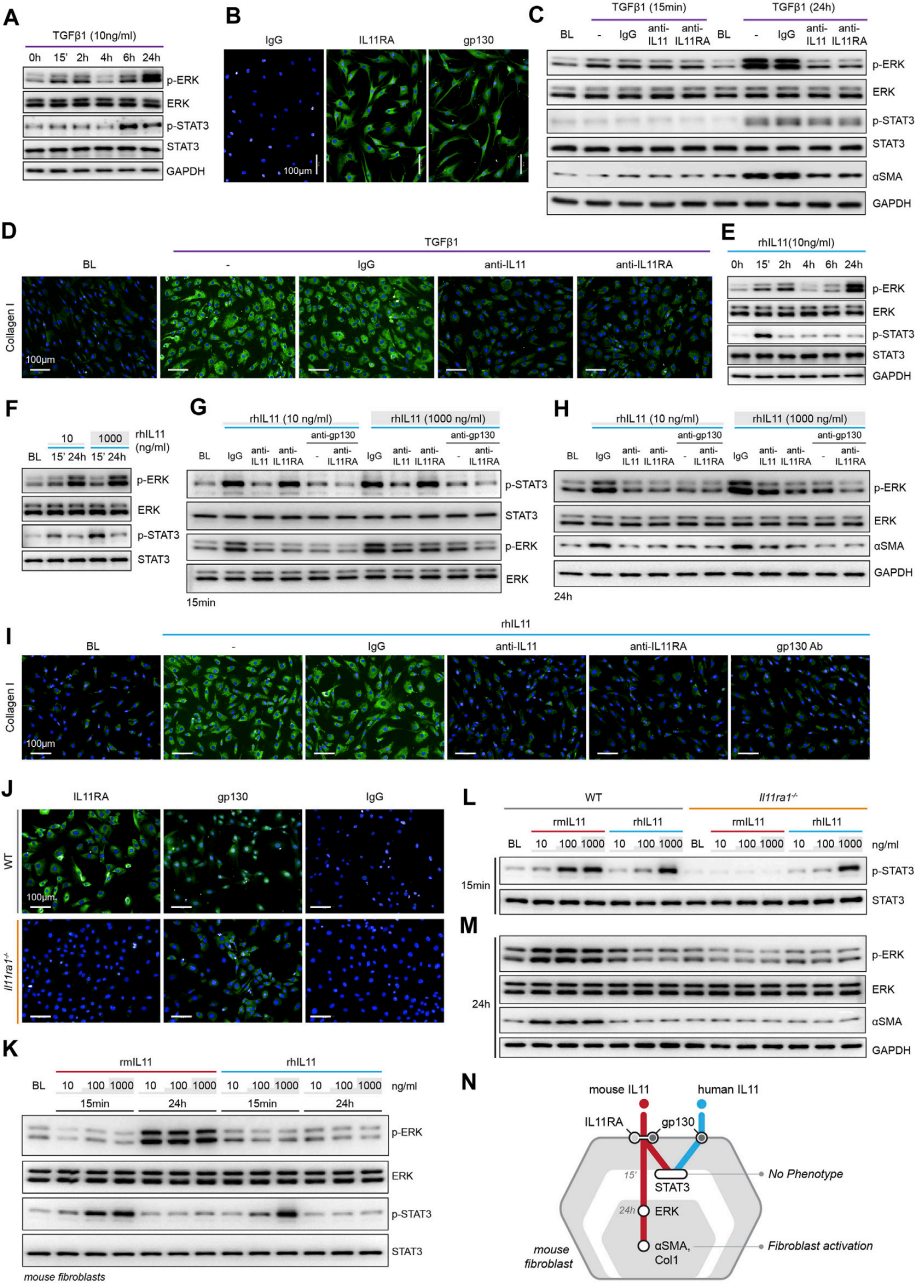
TGFβ1-/IL11-driven fibrogenesis is coincident with activation of ERK, unrelated to STAT3 phosphorylation and shows species-specific effects. **(A)** Western blots of ERK and STAT3 activation in TGFβ1-stimulated HCFs over a time course (*n* = 1). **(B)** Representative immunofluorescence (IF) images of IL11RA, and gp130 expression in HCFs (scale bars, 100 μm; *n* = 3). **(C,D) (C)** Western blot analysis of p-ERK, ERK, p-STAT3, STAT3, and ⍺SMA (*n* = 1) and **(D)** IF images of Collagen I staining (*n* = 3) in TGFβ1-stimulated HCFs in the presence of either IgG, anti-IL11, or anti-IL11RA. **(E)** Western blots of ERK and STAT3 activation status in IL11-stimulated HCFs over a time course (*n* = 1). **(F)** Western blots showing ERK and STAT3 activation status in HCFs following stimulation with low and high dose IL11 (*n* = 1). **(G–I)** Effects of anti-IL11, anti-IL11RA, or anti-gp130 on inhibiting **(G–H)** ERK and STAT3 activation (*n* = 1) and **(I)** Collagen I Induction (*n* = 3) in IL11-stimulated HCFs. **(J)** IF images (scale bars, 100 µm) of IL11RA and gp130 in MCFs isolated from wild-type (WT) and *Il11ra1* ^−/−^ mice (*n* = 3). **(K–M)** Dose-dependent effects of rmIL11 and rhIL11 on **(K)** ERK and STAT3 activation status in wild-type (WT) MCFs, on **(L)** STAT3 activation (15 m) and **(M)** ERK activation and ⍺SMA expression (24 h) in WT and *Il11ra1* ^−/−^ MCFs (*n* = 1). **(N)** Schematic showing the effects of rmIL11 or rhIL11 on signaling and fibroblast activation in mouse fibroblasts. **(A–I)** primary HCFs; 24 h, **(J–M)** primary MCFs, **(A–M)** IL11/TGFβ1 (10 ng/ml), unless otherwise specified. BL: Baseline. Inhibition of STAT3 activity results in ER stress causing fibroblast dysfunction and death.

We profiled primary human cardiac fibroblasts (HCFs) stimulated with TGFβ1 over a 24 h period and observed rapid and sustained phosphorylation of SMAD2, bi-phasic activation of ERK (pERK), and late activation of STAT3 (pSTAT3) (Figure 1A; Supplementary Figure S1A). HCFs progressively secreted IL11 over a time course following TGFβ1 stimulation, with marked upregulation by 24 h (Supplementary Figure S1B). Immunofluorescence (IF) staining revealed coexpression of IL11RA and gp130 in early passage of HCFs, consistent with autocrine IL11 signaling in HCFs (Figure 1B).

To study the functional relevance of TGFβ1-or IL11-induced ERK and/or STAT3 activation, we stimulated HCFs with TGFβ1 in the presence of neutralizing IL11 or IL11RA antibodies that were developed to inhibit fibroblast activation (*in vitro*) and fibrosis phenotypes (*in vivo*), agnostic of the underlying pathways (Widjaja et al., 2019; Widjaja et al., 2021). In TGFβ1-stimulated HCFs, inhibition of IL11 signaling using either anti-IL11 or anti-IL11RA prevented ERK phosphorylation (at the 24 h time point) but had no effect on STAT3 activity (Figure 1C). Downregulation of ERK activity by anti-IL11 or anti-IL11RA was coincident with a reduction in TGFβ1-stimulated fibroblast-to myofibroblast transformation, as evident from lesser ⍺SMA and Collagen expression (Figures 1C,D; Supplementary Figures S1C–E).

IL11-stimulated HCFs exhibited ERK activation, which mirrored the effect seen with TGFβ1 (Figure 1E). IL11 also activated STAT3 in HCFs but its phosphorylation pattern differed from TGFβ1 with an early (15 m) increase followed by progressively lower levels that returned to baseline by 24 h. We then stimulated HCFs with physiological (10 ng/ml) or supra-physiological (1,000 ng/ml) doses of IL11: ERK was maximally activated with physiological IL11 levels, whereas STAT3 phosphorylation was more pronounced at very high IL11 concentration, questioning the specificity of IL11-stimulated pSTAT3 (Figure 1F).

We then studied the effects of anti-IL11 or anti-IL11RA as compared to a commercial anti-IL11 (MAB218), which is known to inhibit cardiac fibroblast-to-myofibroblast transformation (Schafer et al., 2017), as well as a gp130-neutralizing clone (Supplementary Figure S1F). IL11-induced ERK phosphorylation was similarly inhibited by all four neutralizing antibodies (Figures 1G–H; Supplementary Figure S1G). In contrast, MAB218 or anti-IL11RA reduced only ERK phosphorylation but did not prevent STAT3 phosphorylation (Figures 1G,H; Supplementary Figures S1G–H). In IL11 stimulated HCFs, anti-IL11 (two separate clones), anti-IL11RA, and anti-gp130 equally inhibited fibrogenesis as compared to control, as evident from ⍺SMA and Collagen expression levels (Figures 1H,I; Supplementary Figures S1I–K).

These studies show that both TGFβ1 and IL11 stimulate fibroblast activation and ERK phosphorylation, but they differentially affect STAT3. Furthermore, while neutralizing antibodies against IL11, IL11RA or gp130 all inhibit fibroblast activation and ERK signaling, only some antibodies inhibit STAT3 phosphorylation. These data suggest that STAT3 activation is not directly related to fibroblast activation.

### IL11RA1-independent Effects of High Concentration Human IL11 in Mouse Fibroblasts

There are discrepancies in the literature relating to the downstream mediators of IL11, which may relate to the use of recombinant human IL11 (rhIL11) in mouse experiments (Cook and Schafer, 2020). We stimulated primary mouse cardiac fibroblasts (MCFs), which co-express IL11RA1 and gp130 (Figure 1J), with either rhIL11 or recombinant mouse IL11 (rmIL11) over a dose range (ng/ml: 10, 100, 1,000) for 15 m or 24 h. In MCFs, species-matched rmIL11 activated ERK activation with maximal phosphorylation observed at the lowest concentration tested (10 ng/ml). rmIL11 also induced STAT3 activation with most notable effects at very high IL11 concentration (1000 ng/ml) (Figure 1K). In contrast, species-unrelated rhIL11 did not activate ERK in MCFs but instead induced STAT3 phosphorylation when used at high concentrations (≥100 ng/ml) (Figure 1K).

We studied the species-specific effects in more detail using rhIL11 or rmIL11 on MCFs isolated from *Il11ra1* null mice (*Il11ra1* ^*−/−*^) or wild-type (WT) controls. rmIL11 resulted in STAT3 phosphorylation in WT MCFs at higher doses (>100 ng/ml) but did not activate STAT3 in *Il11ra1* ^*−/−*^ cells (Figure 1L). In WT cells, rmIL11 induced ERK activation and ⍺SMA upregulation at low concentration (10 ng/ml), but had no effect on pERK, pSTAT3 of fibrogenesis in *Il11ra1* ^*−/−*^ MCFs (Figure 1M). In contrast, rhIL11 activated STAT3 in both *Il11ra1* ^*−/−*^ and WT fibroblasts when used at high concentrations but had no effect on ERK activation (Figures 1L,M). Irrespective of its effects on pSTAT3, rhIL11 did not stimulate activation of fibroblasts in MCFs of any genotype.

Overall, these data show unexpected effects of species-unrelated rhIL11 in MCFs. While rhIL11 does not induce pERK or stimulate fibrogenesis in MCFs, high concentrations of rhIL11 activate STAT3 in both WT and *Il11ra1* ^*−/−*^ fibroblasts. This suggests direct binding of high dose rhIL11 to mouse gp130, independent of IL11RA1, an effect that is not associated with fibrogenesis (Figure 1N).

**FIGURE 2:**
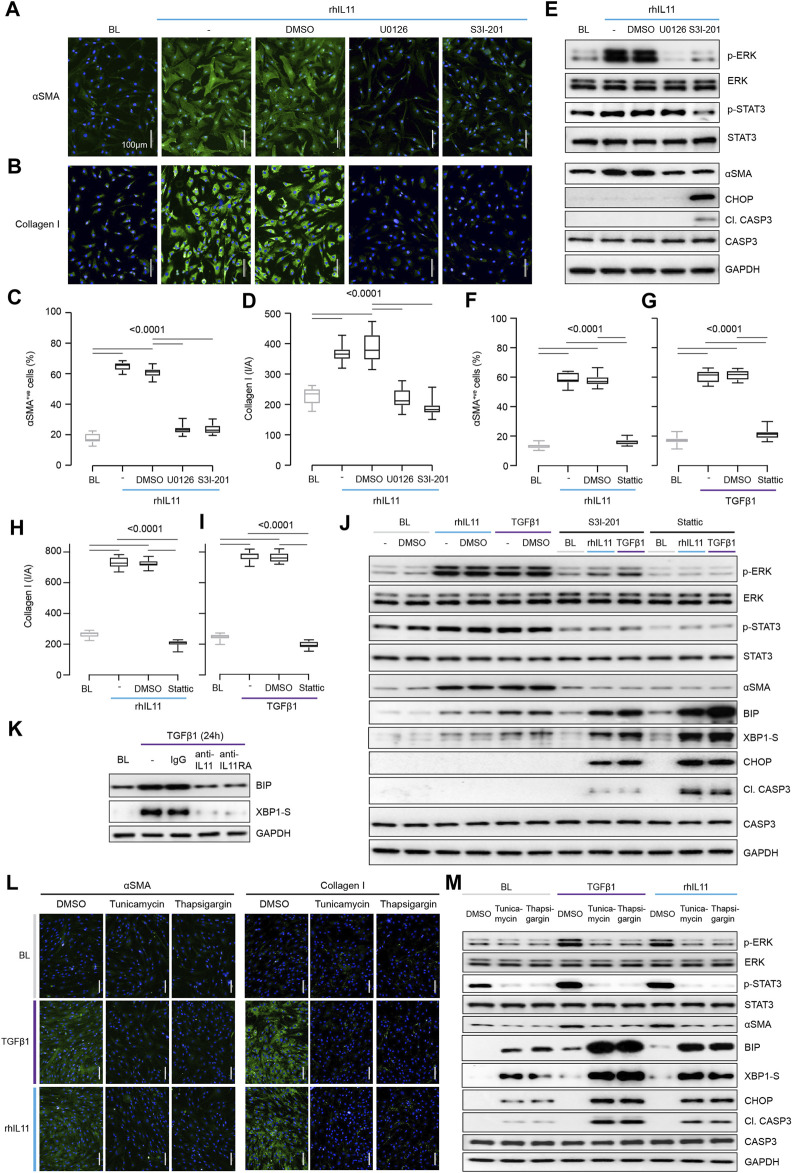
Inhibition of STAT3 in TGFβ1-or IL11-stimulated fibroblasts causes ER stress-related fibroblast dysfunction mimicking effects of generic ER stressors. **(A–E)** Effects of U0126 or S3I-201 on rhIL11-stimulated HCFs. **(A,B)** IF images (scale bars, 100 μm; *n* = 3) and **(C,D)** quantification of ⍺SMA^+ve^ cells and Collagen I immunostaining (*n* = 14). **(E)** Western blots showing ERK, STAT3, and Caspase3 activation status and ⍺SMA and CHOP protein expression (*n* = 1). **(F–I)** Quantification of **(F,G)** ⍺SMA^+ve^ cells and **(H,I)** Collagen I immunostaining following stimulation with **(F, H)** rhIL11 or **(G, I)** TGFβ1 in the presence of Stattic (*n* = 14). **(J)** Effects of S3I-201 or Stattic on the activation of ERK, STAT3, and Caspase3 and on the expression of ⍺SMA, CHOP, and XBP1-S at baseline and in TGFFβ1-or IL11-stimulated HCFs (*n* = 1). **(K)** Western blots of BIP and XBP1-S from IgG/anti-IL11/anti-IL11RA-treated TGFβ-stimulated HCFs (*n* = 1). **(L,M) (L)** Representative IF images (scale bars, 100 µm) of ⍺SMA^+ve^ cells and Collagen I (*n* = 3) and **(M)** Western blot analysis of pERK, ERK, pSTAT3, STAT3, ⍺SMA, BIP, XBP1-S, CHOP, Cleaved Caspase3, Caspase3, and GAPDH from TGFβ1-or rhIL11-stimulated HCFs (*n* = 1). **(A–M)** primary HCFs; 24 h; rhIL11/TGFβ1 (10 ng/ml), U0126 (10 µM), S3I-201 (20 µM), Stattic (2.5 µM), IgG/anti-IL11/anti-IL11RA (2 μg/ml), Tunicamycin (5 μg/ml), Thapsigargin (300 nM). **(C,D, F–I)** Data are shown as box-and-whisker with median (middle line), 25th–75th percentiles (box) and min-max percentiles (whiskers); one-way ANOVA with Tukey’s correction. BL: Baseline. TGFβ1-stimulated protein synthesis is IL11-/ERK- and mTOR-dependent.

To dissect matters further, we examined the effects of pharmacologic inhibition of either ERK (U0126) or STAT3 (S3I-201) in fibroblasts stimulated with IL11. Surprisingly, inhibition of STAT3 was equally effective in preventing fibrogenesis as ERK inhibition (Figures 2A–D). This was unexpected given our earlier findings (Figure 1) but consistent with the literature (Dees et al., 2012; Chakraborty et al., 2017). However, while U0126 inhibited IL11-induced ERK but not STAT3 phosphorylation, S3I-201 inhibited both STAT3 and ERK activation, which should not occur with selective inhibition (Figure 2E).

STAT3 activity has been associated with endoplasmic reticulum (ER) stress that occurs with proteotoxicity (Song et al., 2020). We thus examined ER stress in IL11 stimulated HCFs and found that ERK inhibition with U0126 reduced ⍺SMA induction in the absence of proapoptotic ER stress (CHOP induction and Caspase 3 cleavage) (Figure 2E). In contrast, S3I-201 inhibited both ERK and STAT3 and induced proapoptotic ER stress, which was associated with lesser ⍺SMA expression (Figure 2E). Informed by dose-finding experiments (Supplementary Figure S2A), we used a second STAT inhibitor (Stattic) (Schust et al., 2006). Stattic, like S3I-201, prevented TGFβ1 or IL11-induced fibrogenesis (Figures 2F–I; Supplementary Figure S2B) and caused cell death (Supplementary Figure S2C).

Fibroblasts stimulated with pro-fibrotic factors synthesize and secrete large amounts of extracellular proteins that causes a degree of ER stress, which is compensated for by specific chaperone proteins (Baek et al., 2012; Maiers et al., 2017). In keeping with this, the adaptive ER stress proteins XBP1-S and BIP were mildly upregulated in TGFβ1 or IL11-stimulated HCFs (Figure 2J). However, in the presence of S31-201 or Stattic, TGFβ1 or IL11 resulted in much more severe and pro-apoptotic ER stress (Figure 2J).

As anti-IL11 or anti-IL11RA inhibit IL11-dependent pro-fibrotic protein translation, these antibodies should limit physiological proteotoxic ER stress in HCFs stimulated with pro-fibrotic factors. Indeed, we found that anti-IL11 or anti-IL11RA lowered BIP and XBP1-S expression (Figure 2K) in TGFβ1-stimulated HCFs, thus lowering proteotoxic ER stress overall (Supplementary Figure S2D).

The relationship between ER stress and fibrogenesis was studied further using the generic ER stress activators (thapsigargin or tunicamycin), which robustly inhibited TGFβ1-induced fibrogenesis (⍺SMA and Collagen I expression) (Figures 2L,M; Supplementary Figures S2E,F). When given alone, thapsigargin or tunicamycin caused ER stress, as evidenced by elevated BIP, XBP1-S, CHOP and Caspase 3 cleavage, which was increased further with ER protein loading (e.g., with collagen) following TGFβ1 or IL11 stimulation (Figure 2M). Interestingly, these generic ER stressors reduced pSTAT3 levels below baseline when used alone or in combination with TGFβ1 or IL11 (Figure 2M; Supplementary Figure S2G).

These data show that inhibition of IL11-dependent ERK signaling in TGFB1 stimulated fibroblasts reduces pro-fibrotic gene translation and ER stress. In contrast, inhibition of STAT3 activation causes severe ER stress resulting in fibroblast dysfunction and cell death, which non-specifically prevents myofibroblast transformation.

**FIGURE 3:**
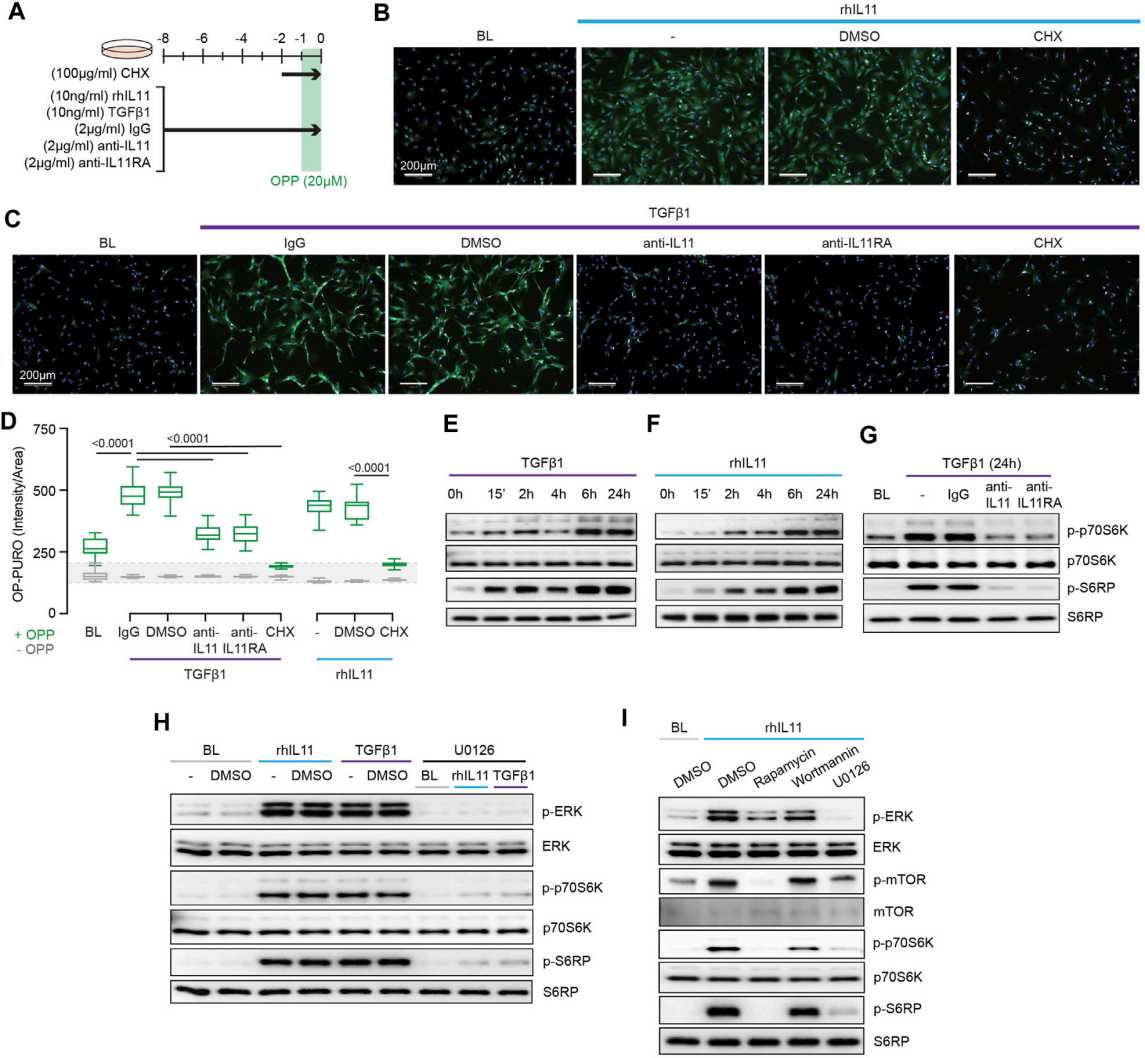
TGFβ1-and IL11-stimulated protein synthesis is ERK- and mTOR-dependent. **(A)** Schematic for experiments shown in **(B–D)**. **(B–D) (B,C)** Immunofluorescence images (scale bars, 100 μm; *n* = 3) and **(D)** quantification of Alexa Fluor™ 488 - OPP signal in HCFs (*n* = 14) following treatments shown in **(A)**. **(E–G)** Western blots of p-p70S6K (T389), p70S6K, p-S6RP, S6RP from (E) TGFβ1 or (F) rhIL11-stimulated HCFs over a time course and on **(G)** IgG, anti-IL11, or anti-IL11RA-treated TGFβ1-stimulated HCFs (*n* = 1). **(H)** Effects of U0126 on TGFβ1 or rhIL11-induced ERK, p70S6K (T389), and S6RP activation (*n* = 1). **(I)** Comparison effects of Rapamycin, Wortmannin, and U0126 on rhIL11-induced ERK, mTOR, p70S6K, and S6RP activation (*n* = 1). **(B–I)** primary HCFs; IL11/TGFβ1 (10 ng/ml), OPP (20 µM), CHX (100 μg/ml), IgG/anti-IL11/anti-IL11RA (2 μg/ml), U0126 (10 µM), Rapamycin (10 nM), Wortmannin (1 µM); **(B–D)** 8 h, **(G–I)** 24 h. **(D)** Data are shown as box-and-whisker with median (middle line), 25th–75th percentiles (box) and min-max percentiles (whiskers); one-way ANOVA with Tukey’s correction. IL11 stimulates translation of proline-rich pro-fibrotic genes *via* EPRS.

We next examined protein synthesis using the OP-Puro Protein Synthesis Assay (Figure 3A). IL11 alone was sufficient to induce protein synthesis in HCFs (Figure 3B). Moreover, TGFβ1-stimulated protein synthesis was IL11-dependent and addition of either anti-IL11 or anti-IL11RA was equally effective in inhibiting this (Figures 3C,D).

Activation of p70S6K is a signaling convergence for canonical protein synthesis pathways that include MEK/ERK and PI3K, among others (Wang et al., 2001). Stimulation of HCFs with TGFβ1 resulted in biphasic phosphorylation of p70S6K and of its downstream target S6 ribosomal protein (S6RP) (Figure 3E). The effects of IL11 stimulation differed slightly with progressive phosphorylation of p70S6K and S6RP over the time course with maximal activation at 24 h (Figure 3F). We examined whether TGFβ1-induced p70S6K activation was IL11-dependent, which proved to be the case (Figure 3G).

The central importance of ERK activation for TGFβ1-or IL11-induced p70S6K activity was apparent from experiments using the MEK inhibitor U0126 (Figure 3H). To explore the pathway components between ERK and p70S6K, we inhibited mTOR with rapamycin and compared its effects with wortmannin. Wortmannin had no effect on p70S6K or S6RP phosphorylation, ruling out a role for PI3K/AKT. In contrast, rapamycin inhibited phosphorylation of mTOR, as expected, and also p70S6K and S6RP activation (Figure 3I), similar to effects seen with U0126 (Figure 3H). This places mTOR activation downstream of IL11-induced MEK/ERK phosphorylation and upstream of p-p70S6K.

IL11 promotes the translation of pro-fibrotic ECM proteins and of itself but does not increase translation of all proteins (Schafer et al., 2017; Cook and Schafer, 2020). Glutamyl-prolyl-tRNA synthetase (EPRS) is important for the specificity of TGFβ1-stimulated translation of proline-rich proteins, such as collagen, in cardiac fibroblasts (Wu et al., 2020). It is also known that EPRS is phosphorylated by p70S6K that can have non-canonical effects on EPRS function (Arif et al., 2017). This prompted us to examine whether EPRS has a role in IL11-stimulated protein synthesis.

**FIGURE 4:**
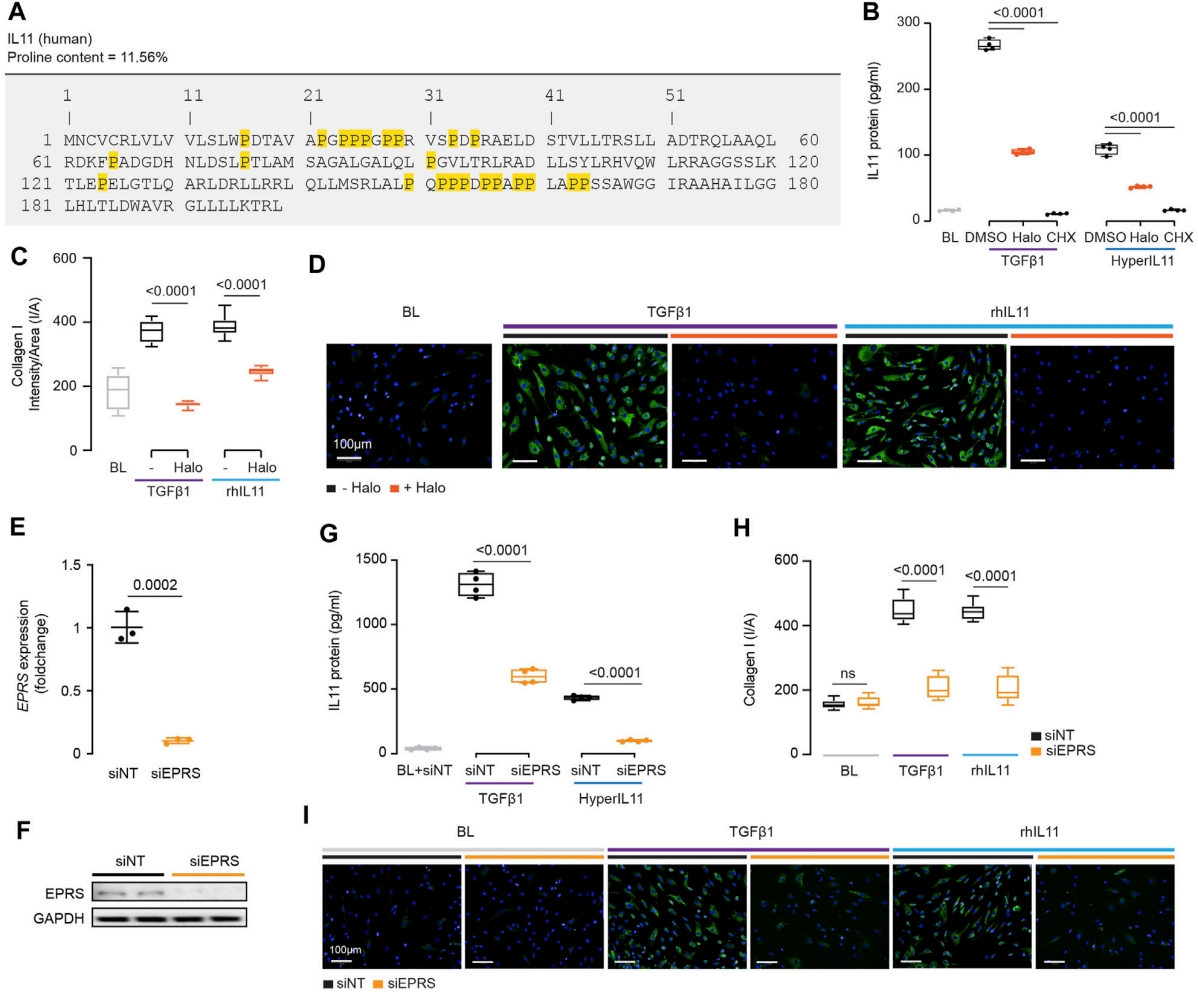
IL11 promotes the translation of proline-rich pro-fibrotic genes, including itself, through EPRS activity. **(A)** Amino acid sequence of human IL11, proline residues highlighted in yellow. **(B)** IL11 levels in the supernatant following TGFβ1 or HyperIL11 stimulation in the presence of Halofuginone (Halo) or Cycloheximide (CHX) (*n* = 3). **(C,D)** **(C)** Quantification (*n* = 14) and **(D)** immunofluorescence images (scale bars, 100 μm; n = 3)) of Halofuginone-treated TGFβ1/IL11-stimulated HCFs for Collagen I staining. **(E,F) (E)** *EPRS* mRNA (*n* = 3) and **(F)** EPRS protein (*n* = 2) expression levels after knockdown using siEPRS. **(G–I) (G)** IL11 levels in the supernatant (*n* = 4), **(H)** quantification (n = 14), and **(I)** immunofluorescence images (scale bars, 100 μm; *n* = 3) of Collagen I in stimulated HCFs subjected to siEPRS. **(B–I)** primary HCFs; IL11/TGFβ1/HyperIL11 (10 ng/ml), Cycloheximide (CHX, 100 μg/ml), Halofuginione (100 nM), non-targeting siRNA (siNT)/EPRS siRNA (siEPRS) (12.5 nM); 24 h. **(B,C, G,H)** Data are shown as box-and-whisker with median **(middle line)**, 25th–75th percentiles (box) and min-max percentiles (whiskers); one-way ANOVA with Tukey’s correction; **(E)** Data are shown as mean ± SD, 2-tailed *t*-test. Signaling relationships between nintedanib, pirfenidone and anti-IL11.

IL11 stimulates its own translation in an autocrine loop and if this were related to EPRS activity then IL11 would need to be proline-rich itself. Examination of human and mouse IL11 revealed a high proline content of both molecules, 11.6 and 8.5% of amino acids, respectively (Figure 4A; Supplementary Figure S3). Of note, there are four PP and three PPP motifs in human IL11, which are also common in collagen, that cause ribosomal stalling and require EPRS and EIF5A activity for continued translation (Doerfel et al., 2013; Mandal et al., 2014).

We incubated HCFs with the EPRS inhibitor halofuginone in the presence of TGFβ1 or an IL11:IL11RA fusion construct (HyperIL11), which is not detected by IL11 ELISA (Schafer et al., 2017), and measured IL11 levels in the supernatant. Halofuginone reduced IL11 secretion downstream of either TGFβ1 or HyperIL11 stimulation (Figure 4B). Collagen has multiple PPG motifs, and we confirmed its induction by TGFβ1 is EPRS-dependent while extending findings to show that IL11-induced collagen secretion also requires EPRS activity (Figures 4C,D).

The specificity of halofuginone inhibition of EPRS is established (Keller et al., 2012) but to further confirm our findings we used siRNA. Knockdown of EPRS in HCFs using silencing RNAs against EPRS (siEPRS) was confirmed at the RNA and protein level, as compared to non-targeting siRNA (siNT) (Figures 4E,F). As seen with halofuginone, siEPRS reduced TGFβ1-or HyperIL11-induced IL11 secretion and collagen production (Figures 4G–I).

Here, we confirmed that collagen synthesis requires EPRS for its translation, identify IL11 as a proline rich molecule containing ribosome stalling motifs and show that EPRS activity underlies IL11 translation.

Pirfenidone and nintedanib are approved drugs for the treatment of fibrotic lung disease but the MOA of these drugs remains poorly understood (Schaefer et al., 2011; Roth et al., 2015). Using insights from the experiments described above, we examined the effects of pirfenidone or nintedanib as compared to anti-IL11 in HCFs or lung fibroblasts stimulated with TGFβ1 to determine if MOAs are related, overlap or distinct.

**FIGURE 5:**
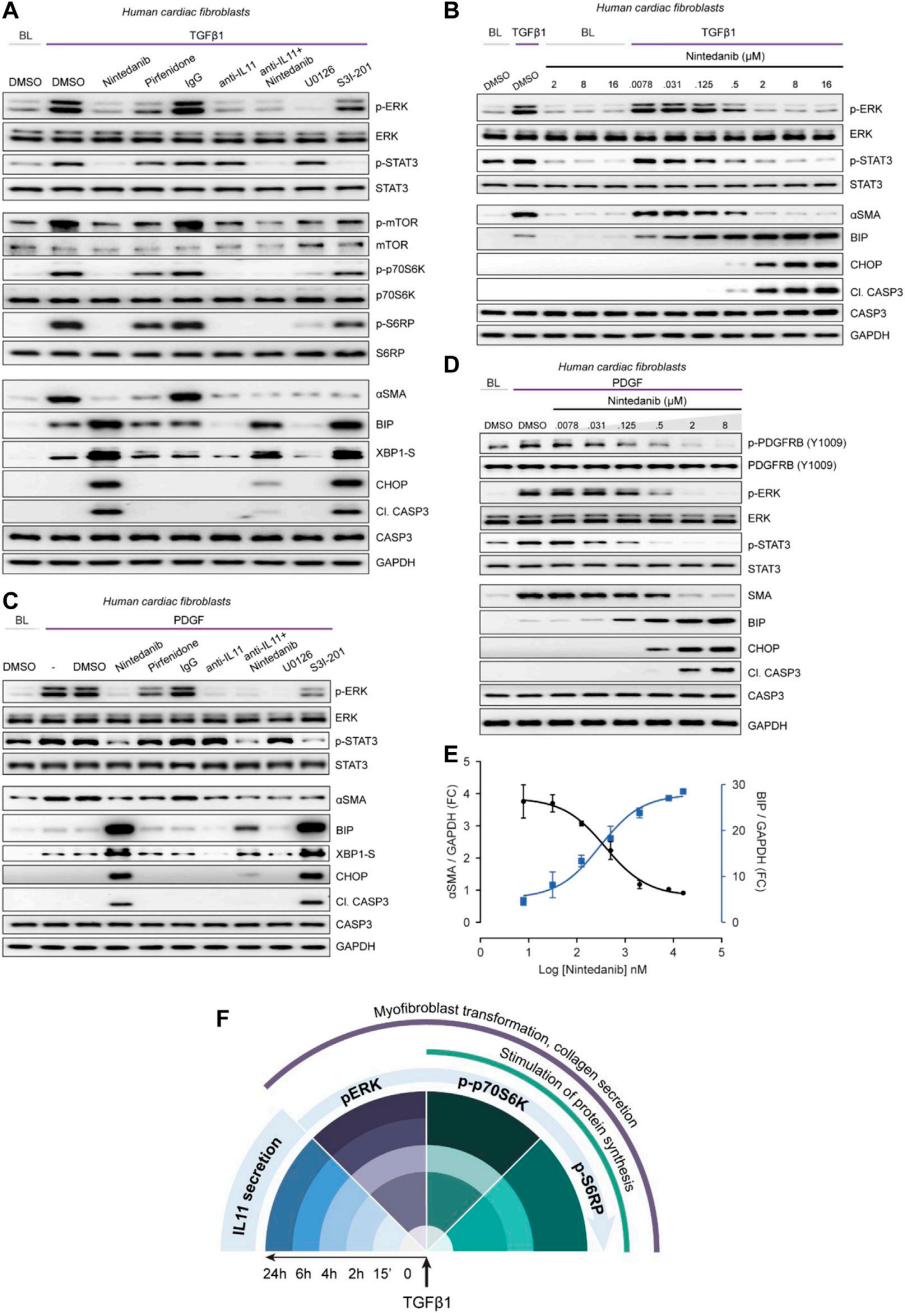
Nintedanib, Pirfenidone and anti-IL11 have different anti-fibrotic mechanisms of action in cardiac fibroblasts. **(A)** Western blots showing activation status of ERK, STAT3, mTOR, p70S6K (T389), S6RP, and Caspase3, and protein expression of ⍺SMA, BIP, XBP1-S, and CHOP following treatment with nintedanib, pirfenidone, IgG, anti-IL11, a combination of nintedanib, anti-IL11, U0126 or S3I-201 from TGFβ1-stimulated HCFs (*n* = 1). **(B)** Western blots of p-ERK, ERK, p-STAT3, STAT3, ⍺SMA, BIP, CHOP, Cleaved Caspase 3, Caspase 3, and GAPDH from HCFs treated with different concentrations of nintedanib in the absence or presence of TGFβ1 (*n* = 1). **(C)** Western blots of p-ERK, ERK, p-STAT3, STAT3, ⍺SMA, BIP, CHOP, Cleaved Caspase 3, Caspase 3, XBP1-S, and GAPDH from PDGF-stimulated HCFs (*n* = 1). **(D)** Western blots of p-PDGFRB, PDGFRB, p-ERK, ERK, p-STAT3, STAT3, BIP, CHOP, Cleaved Caspase 3, Caspase 3, and GAPDH from PDGF-stimulated HCFs treated with different concentrations of nintedanib (*n* = 1). **(E)** Densitometry analysis of ⍺SMA versus BIP expression from TGFβ1-stimulated HCFs treated with different concentrations of nintedanib (*n* = 1). **(F)** Schematic overview and timeline of key pro-fibrotic events that occur downstream of TGFβ1-stimulation in human fibroblasts. **(A–E)** primary HCFs, 24 h; TGFβ1 (10 ng/ml), PDGF (20 ng/ml), IgG/anti-IL11 (2 μg/ml), nintedanib (2 µM), pirfenidone (0.3 mg/ml), U0126 (10 µM), S3I-201 (20 µM), unless otherwise specified.

In TGFβ1-stimulated HCFs, nintedanib reduced pERK to baseline and pSTAT3 below baseline while inhibiting the protein synthesis pathway (p-mTOR, p-p70S6K and pS6RP) and reducing ⍺SMA levels (Figure 5A). Unexpectedly, nintedanib caused ER stress as evidenced by increased BIP, XBP1-S, CHOP, and cleaved Caspase 3 levels, similar to that seen with S31-201 or the ER stressors, thapsigargin and tunicamycin (Figure 2).

Pirfenidone mildly diminished pERK and pSTAT3 and had limited effect on pmTOR, p-p70S6K or pS6RP as compared to nintedanib or anti-IL11. Furthermore, pirfenidone was not associated with increased ER stress over TGFβ1 treatment alone. Anti-IL11 recapitulated earlier findings: inhibiting protein synthesis while lowering TGFβ1-induced proteotoxic ER stress (Figures 2, 3). In addition, anti-IL11 reduced nintedanib-associated ER stress when used in combination (Figure 5A). Nintedanib, a tyrosine kinase inhibitor, is not known to cause ER stress and while the concentration we used (2 µM) is similar to that commonly applied (Wollin et al., 2015), we probed matters further using a dose-response (nintedanib: 16 µM-7.8nM; 4-fold dilutions) (Figure 5B). Nintedanib at a concentration up to 16 µM had no effect on ER stress in quiescent fibroblasts. However, in TGFβ1-stimulated HCFs, nintedanib began to inhibit ⍺SMA expression at 125 nM and complete inhibition was observed at 2 µM. Over the same concentration range, BIP was progressively induced and at the higher end of the range (0.5 and 2 µM), pro-apoptotic ER stress (CHOP/cleaved Caspase 3) upregulation was more apparent. Nintedanib also inhibited pERK and pSTAT in a dose-dependent manner, which was inversely related to the induction of ER stress markers.

Experiments were repeated in TGFβ1-stimulated human lung fibroblasts to exclude organ-of-origin-specific effects in fibroblasts (Supplementary Figure S4). In lung fibroblasts, nintedanib reduced pSTAT3 below baseline, diminished pERK and limited synthesis of ⍺SMA while inducing pro-apoptotic ER stress, which was lesser with co-administration of anti-IL11. Pirfenidone slightly reduced pERK and pSTAT3 and had lesser effects on the protein synthesis pathway than nintedanib or anti-IL11, as seen in HCFs. Pirfenidone reduced ⍺SMA expression, as expected, but had no effect over TGFβ1 on markers of ER stress.

The MOA of Nintedanib is thought primarily through inhibition of PDGF signaling in fibroblasts. We thus examined the effects of Nintedanib on PDGF-stimulated HCF signaling and observed very similar effects to those seen with TGFβ1 stimulation: lesser pSTAT and pERK as well as induction of pro-apoptotic ER stress, which was associated with lesser ⍺SMA induction (Figure 5C). As we did for TGFβ1, we explored the dose-response effect of nintedanib on PDGF signaling, this time also assaying the activation status of PDGFRB (Figure 5D). As expected, there was a dose-dependent decrease in PDGF-stimulated p-PDGFRB but once again there was dose-dependent ER stress which mirrored lesser ⍺SMA induction and was associated with reduced pERK and pSTAT3 (Figure 5D). The relationship between nintedanib dose, ER stress and ⍺SMA induction was formally assessed using semi-quantitative densitometry which revealed dose-dependent reciprocal relationships between ER stress and fibrogenesis (Figure 5E).

## Discussion

In this study, we set out to dissect the signaling pathways by which TGFβ1-induced IL11 activity specifically increases profibrotic gene translation but not transcription. In particular, we wished to determine the relative contributions of ERK from STAT3 downstream of IL11. IL11 is a little studied and somewhat misunderstood cytokine (Cook and Schafer, 2020; Widjaja et al., 2020; Dong et al., 2021), as exemplified by a publication on cardiac fibrosis where it was originally (and erroneously) reported as anti-fibrotic (Obana et al., 2010).

We have speculated that confusion relating to IL11 biology in general, and IL11-related STAT3 phosphorylation in particular, may stem from the use of human IL11 in murine cells and models, as noted previously (Widjaja et al., 2021). Here we show that species unmatched rhIL11 activates STAT3 in both WT and *I11ra1*-deleted MCFs but does not activate ERK, which is IL11RA1 dependent and drives fibrogenesis. Furthermore, neutralizing antibodies against IL11, IL11RA or gp130 all block fibrosis phenotypes and consistently inhibit ERK whereas only some antibodies impact pSTAT3. As such, STAT3 activation in fibroblasts downstream of IL11 stimulation appears uncoupled from fibrosis phenotypes and may represent a bystander event or be related to other phenotypes.

Inhibition of IL11 signaling mimicked the antifibrotic effects seen with the MEK/ERK inhibitor, U0126. We were therefore puzzled to observe that STAT3 inhibition prevented fibrogenesis but noted that S31-201 reduced ERK phosphorylation, which it should not. STAT3 inhibition has been linked with pro-apoptotic ER stress and we explored this possibility in our study (Song et al., 2020). TGFβ1 or IL11 resulted in mild proteotoxic ER stress, as expected, but this homeostatic mechanism transitioned to pro-apoptotic ER stress with STAT3 inhibition. Tellingly, incubation of HCFs with thapsigargin or tunicamycin resulted in severe ER stress and inhibited fibrogenesis in stimulated fibroblasts. Hence, while we confirm that inhibition of STAT3 reduces fibroblast activation, in keeping with the literature (Dees et al., 2012; McHugh, 2017), this effect appears to be ER-stress/cell death related. The mechanism behind this phenomenon requires further study but there are precedents in other contexts (Kimura et al., 2012; Song et al., 2020).

The importance of mTOR signaling for stromal cell activation has been described in cancer-associated fibroblasts, dermal fibroblasts, and hepatic stellate cells (Woodcock et al., 2019). Here, we confirmed that IL11 underlies protein translation in TGFβ1-stimulated HCFs and identified that activation of ERK/mTOR/P70S6K is central to this process (Figure 5F). Whether 1) ERK activates mTOR directly or there is greater upstream complexity, or 2) there are other downstream pathways by which mTOR regulates pro-fibrotic protein synthesis remain to be elucidated. Irrespective of this, our data define an important new intersection between inflammatory and metabolic signaling, which may have implications for immunometabolism and/or inflammaging. The growing appreciation of the linkage between metabolism with fibrosis (fibrometabolism) appears even stronger given the IL11:mTOR interaction (Du et al., 2008; Selvarajah et al., 2021).

Protein synthesis is the most energy-intensive process in growing cells and thus strongly regulated and performed in a selective manner (Buttgereit and Brand, 1995). In fibroblasts, EPRS, the bifunctional glutamate/proline tRNA ligase, specifically controls translation of proline rich ECM genes, such as collagen (Wu et al., 2020). We found that IL11 has an unusually high proline content and encodes motifs associated with ribosome stalling that require EPRS and eIF5A for efficient translation (Doerfel et al., 2013). As with collagen, we found that IL11 requires EPRS, a P70S6K target (Arif et al., 2017), for its translation and secretion, which is thus coordinated with that of ECM proteins during fibrogenesis.

Nintedanib was first developed as an anticancer drug and pirfenidone for inflammation and it was only later that these drugs were found to have anti-fibrotic effects (Schaefer et al., 2011; Roth et al., 2015). Nintedanib is a receptor tyrosine kinase inhibitor, with activity against VEGFR, PDGFR and FGFR with IC50 values of 13–34 nM, 59–65 nM and 37–108 nM, respectively (Hilberg et al., 2008). Nevertheless, in a panel of 33 additional kinases, nintedanib additionally inhibits Flt-3, Lck, Lyn and Src with IC50 values of 26, 16, 196 and 156 nM respectively (Hilberg et al., 2008). The anti-fibrotic MOA of nintedanib remains an issue of debate.

In TGFβ1-or PDGF-stimulated fibroblasts, nintedanib reduced pSTAT3 below baseline, which was accompanied by the induction of pro-apoptotic ER stress. Interestingly, in a recent high-throughput screen to repurpose existing drugs as anti-fibrotics, haloperidol was found to inhibit TGFβ1-induced fibroblast activation and its MOA related to the induction of ER stress (Rehman et al., 2019). The mechanism and directionality by which nintedanib inhibits pSTAT3 and induces ER stress remain to be determined but could relate to activity against, perhaps ER-localised, JAK/STAT. We propose that nintedanib’s anti-fibrotic MOA in TGFβ1-stimulated fibroblasts is due, at least in part, to induction ER stress with effects at concentrations at/or below those that inhibit PDGFR activation.

In conclusion, we studied the pro-fibrogenic, translational-specific signaling activity of IL11 and found an axis of ERK/mTOR/p70S6K activity to be of central importance while discounting a role for STAT3. We suggest that therapeutic inhibition of IL11 might be considered for treating fibrotic diseases using a MOA that is differentiated from approved anti-fibrotic drugs. Given the safety data evident from humans and mice with *IL11RA* or *IL11* loss of function and the lack of toxicities with long term anti-IL11 administration (Widjaja et al., 2019; Ng et al., 2021), it is hoped that IL11-targeting approaches may have lesser side effects than current therapies.

## Data Availability

The original contributions presented in the study are included in the article/Supplementary Material, further inquiries can be directed to the corresponding authors.
