# Supplementary figures and images for: Molecular Dissection of Pro-Fibrotic IL11 Signaling in Cardiac and Pulmonary Fibroblasts
Source: Front Mol Biosci. 2021 Sep 28;8:740650. doi: 10.3389/fmolb.2021.740650 (PMC8505966; doi:10.3389/fmolb.2021.740650)

**1A**

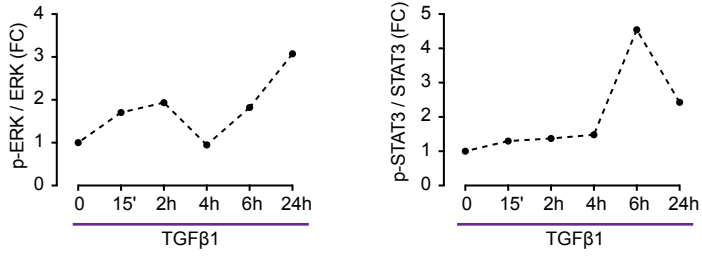

**1C**

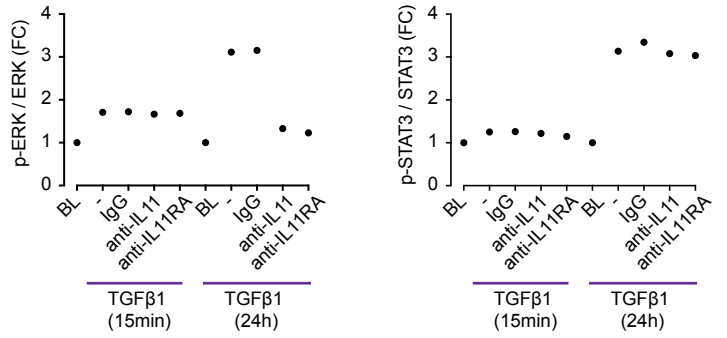

1E

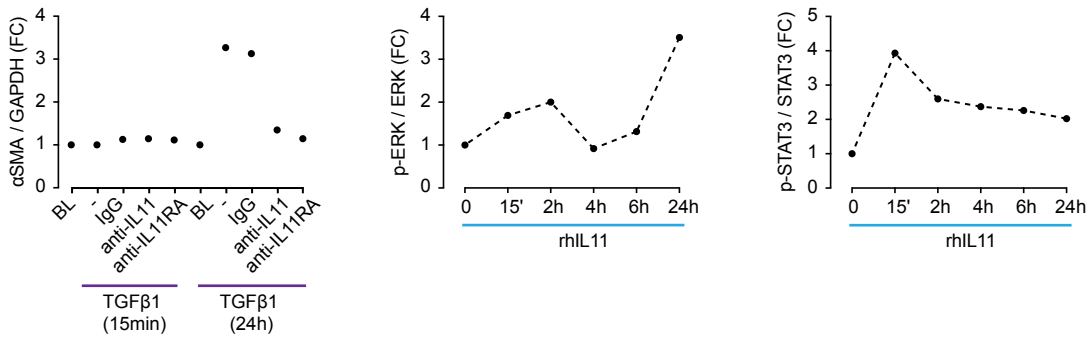

**1F**

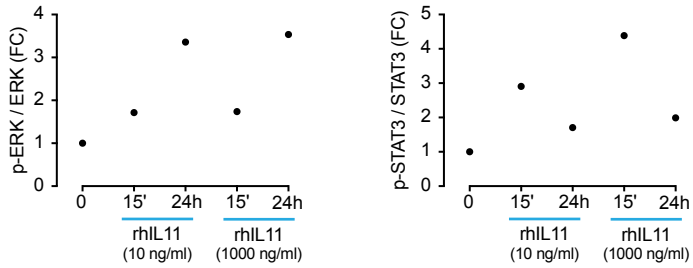

**1G**

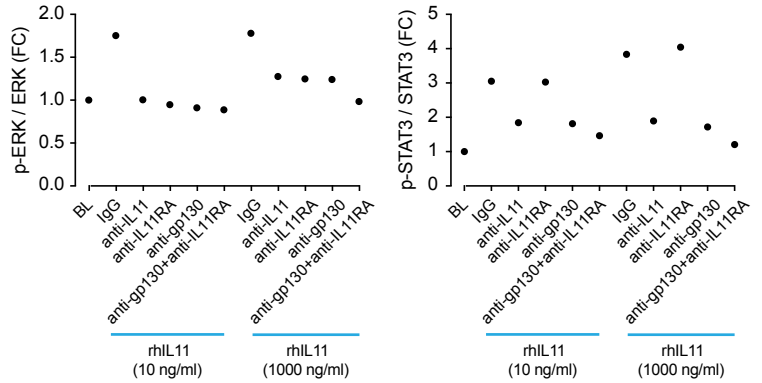

1H

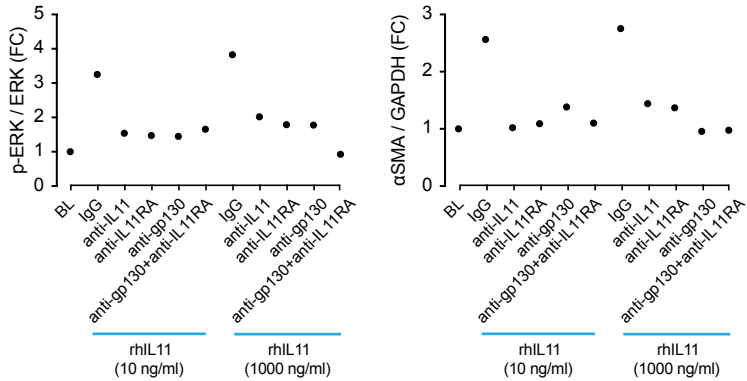

1K

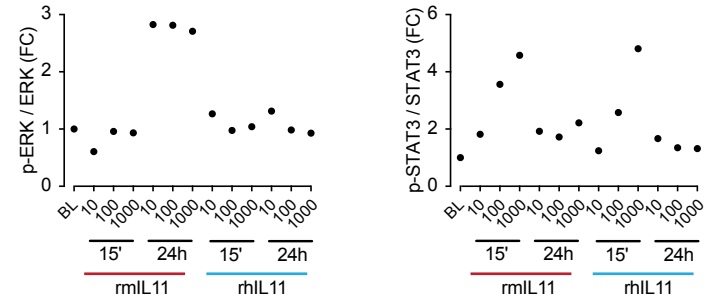

## 1L

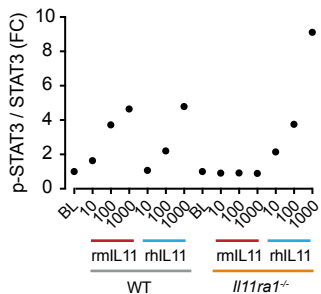

1M

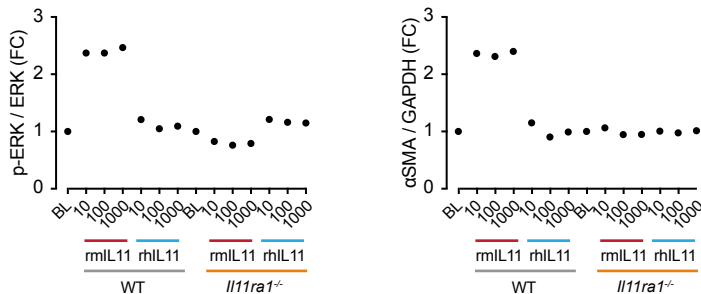

2E

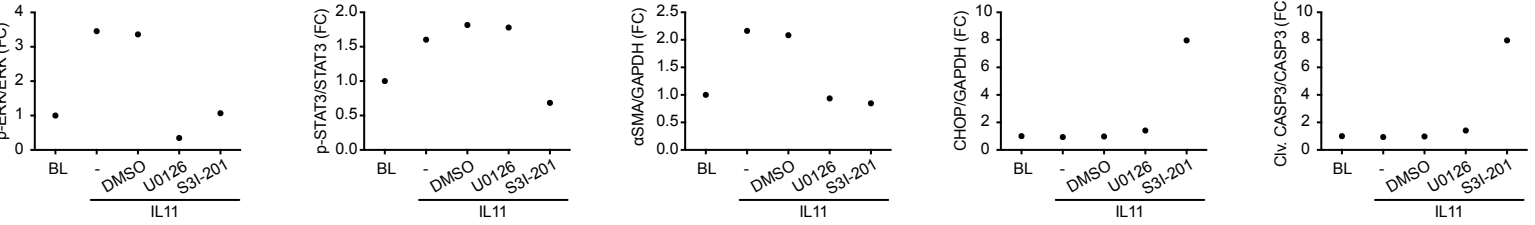

2J

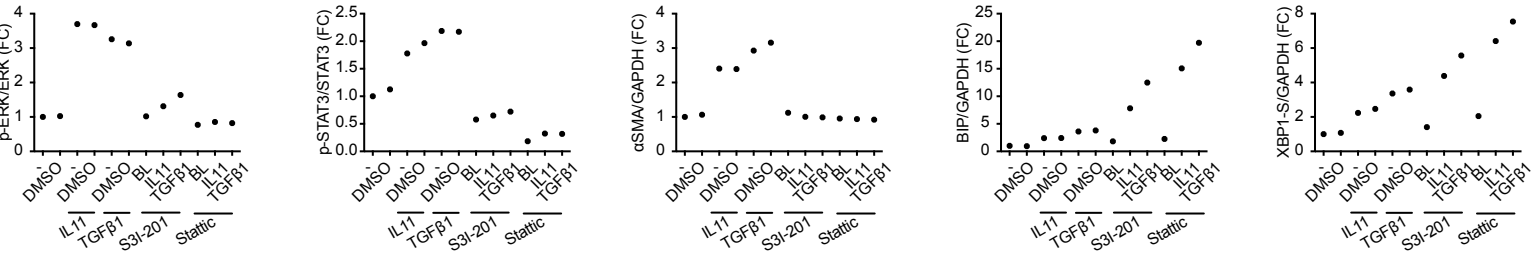

2K

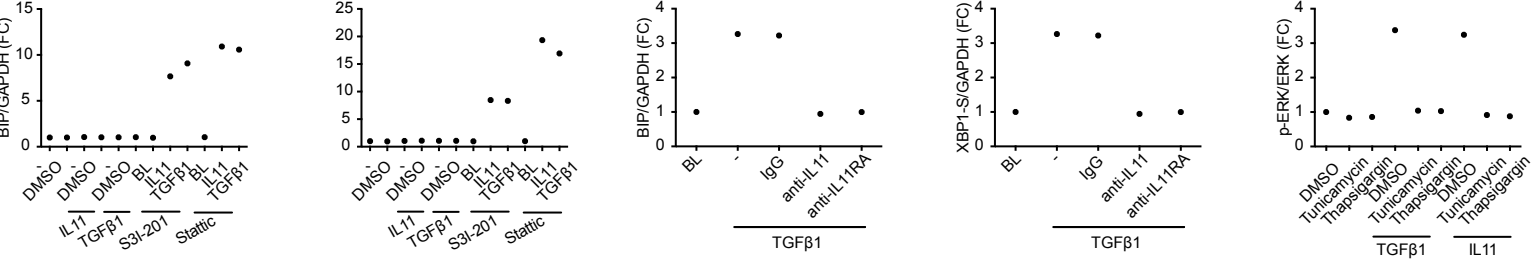

2M

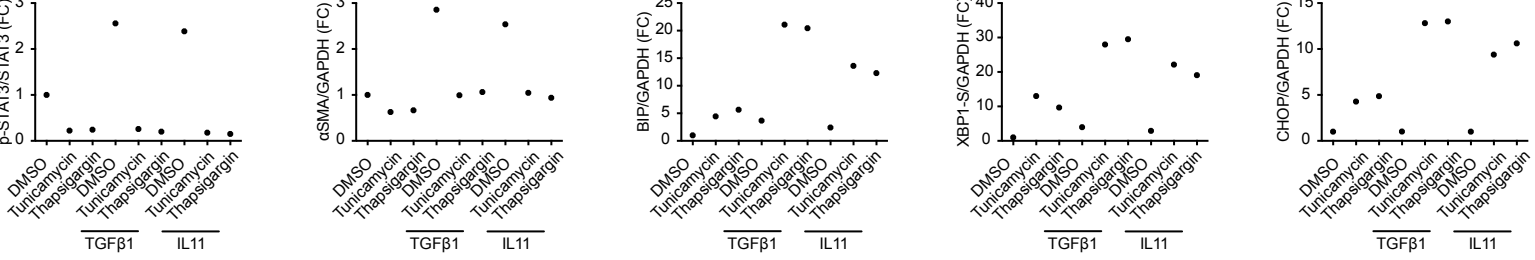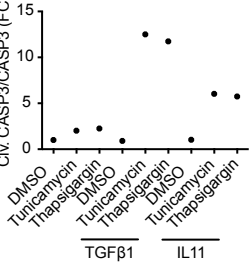

3E

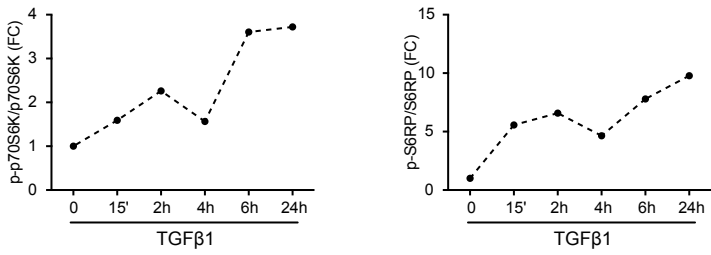

3F

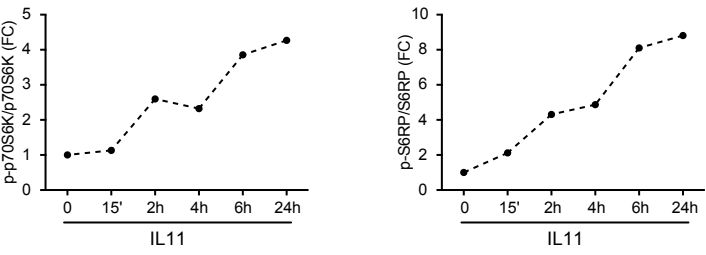

3G

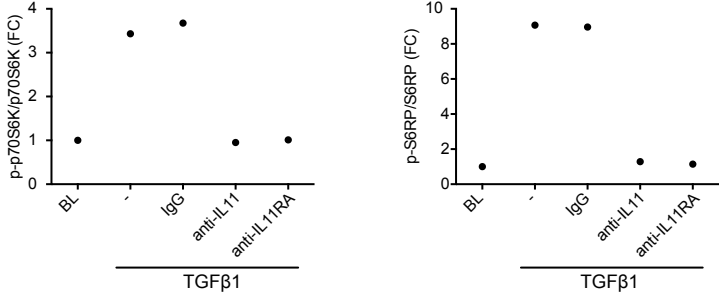

3H

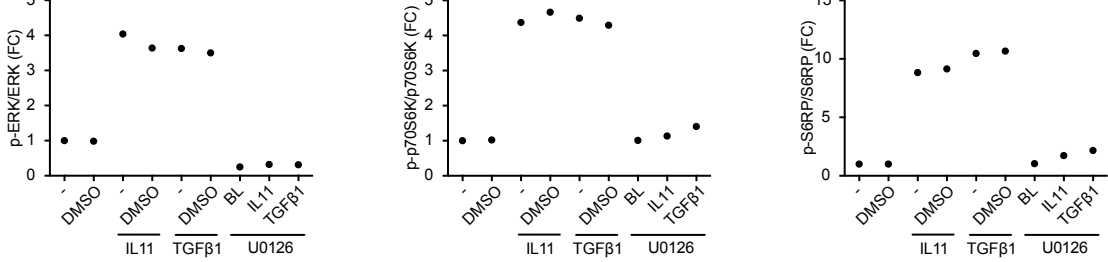

3I

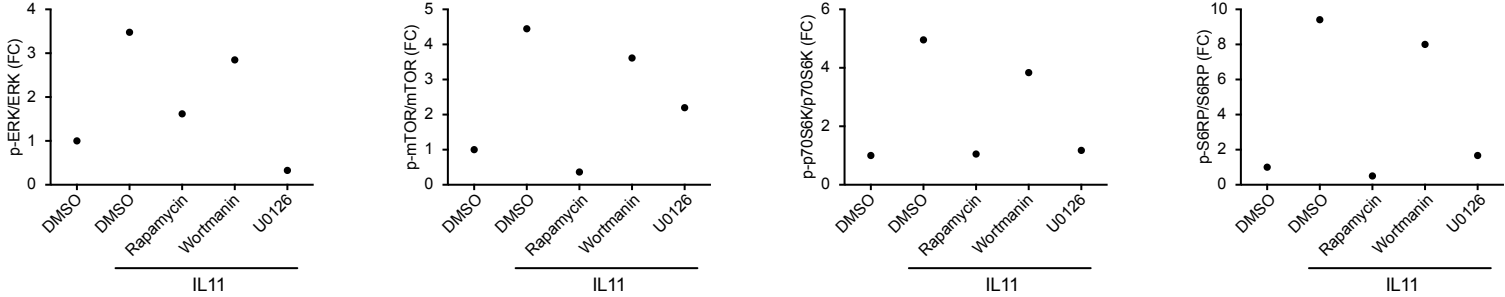

4F

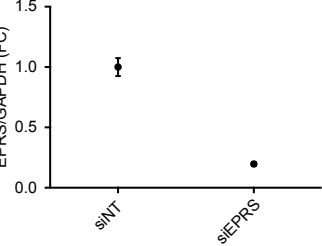



5C

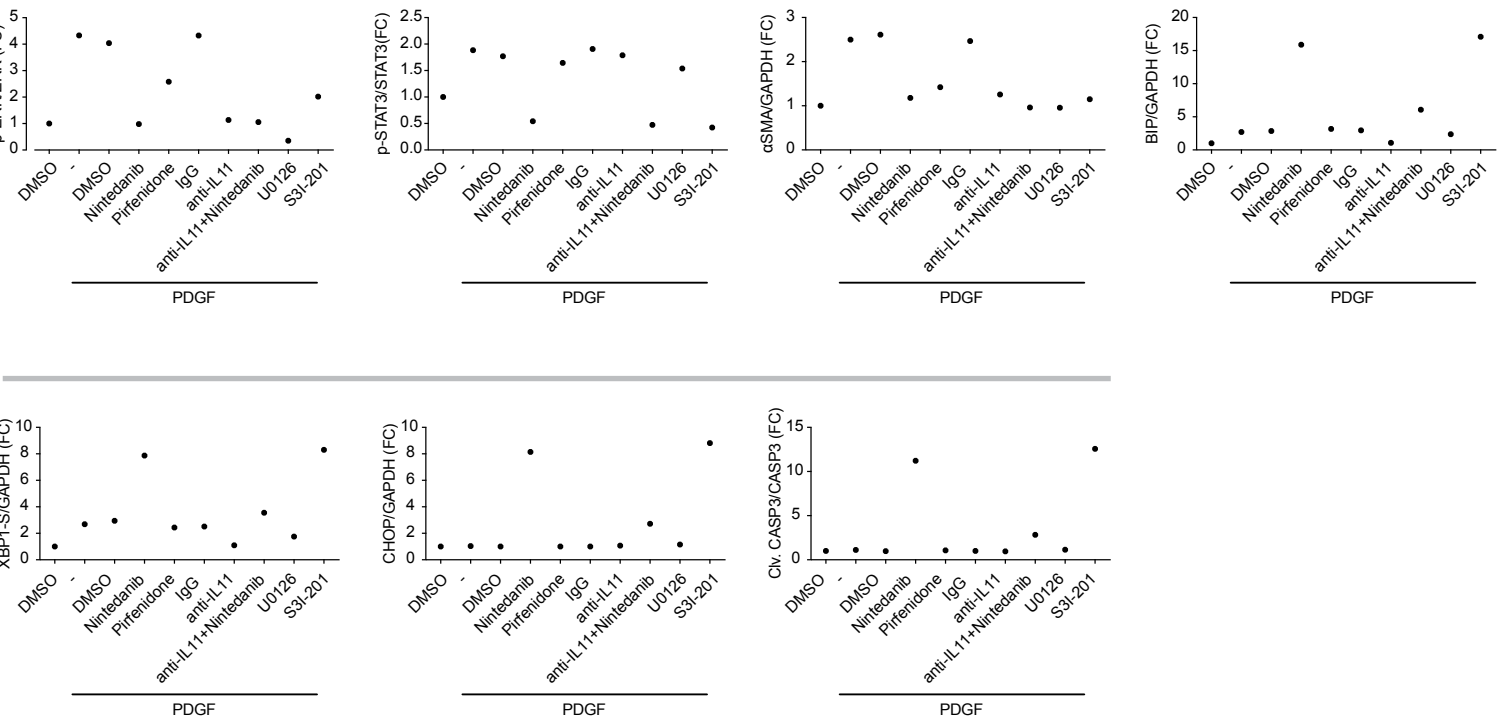

5D

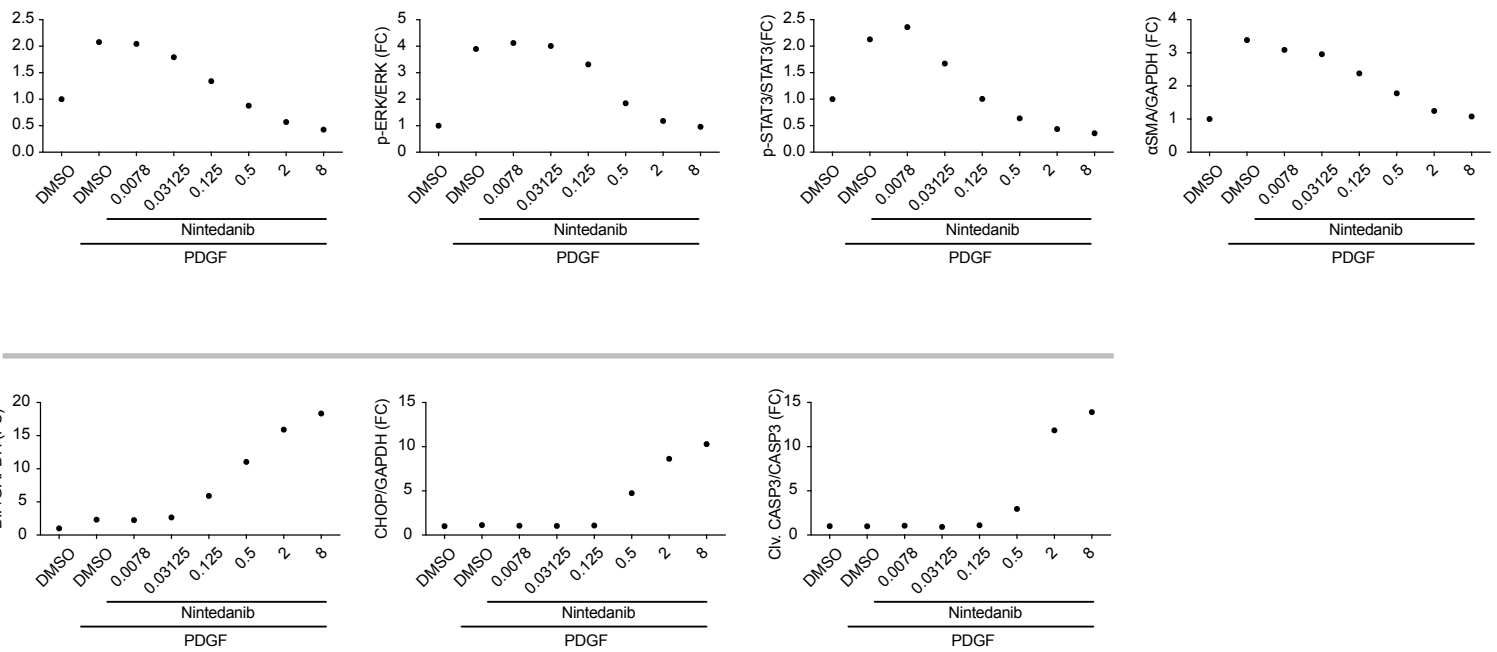

S1A

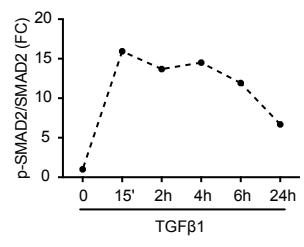

S1G

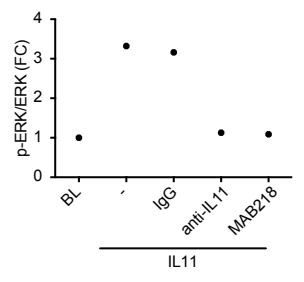

S1H

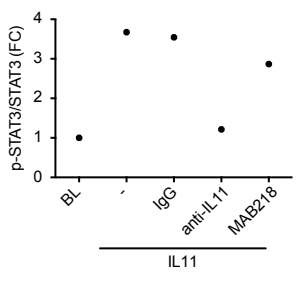

S2A

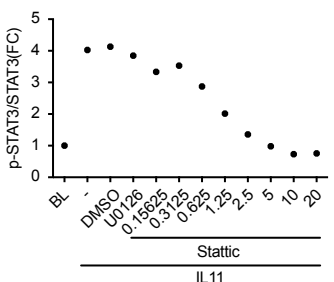

S2D

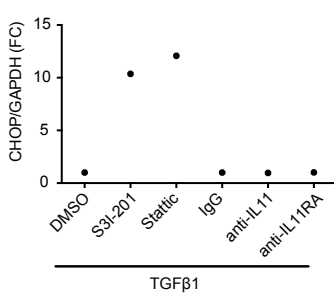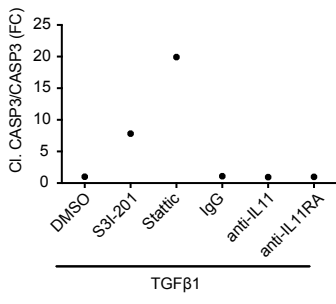

S2G

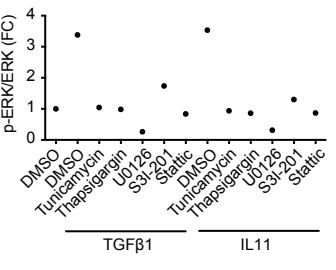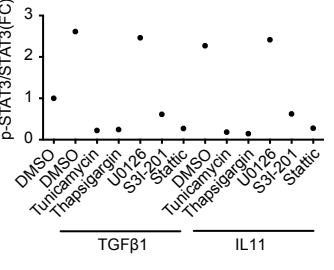

S4

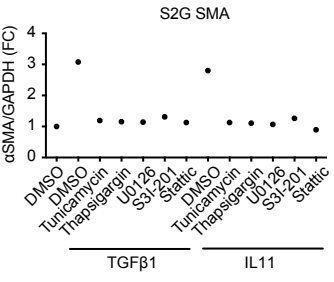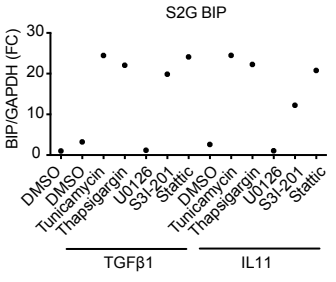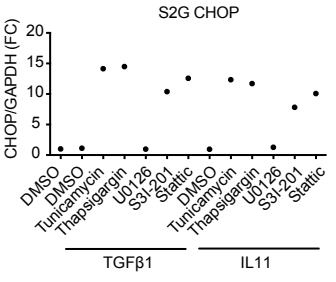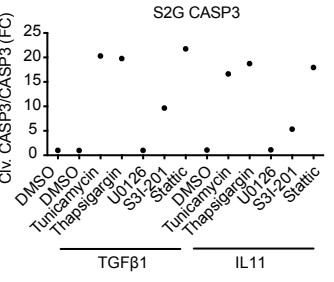

S4

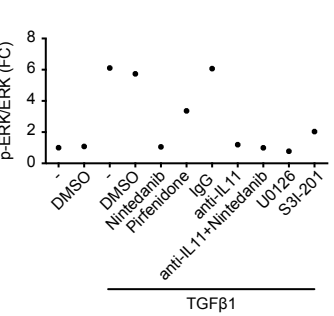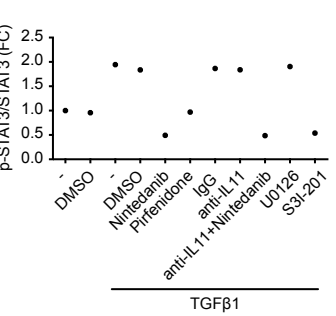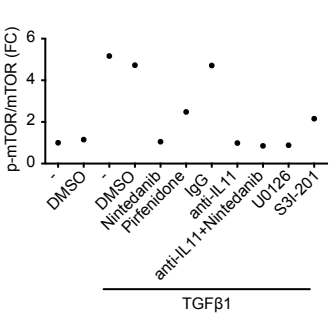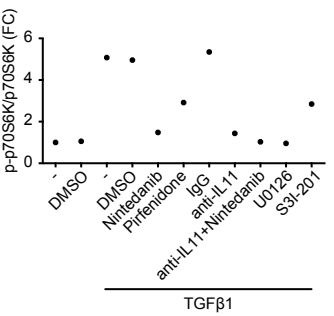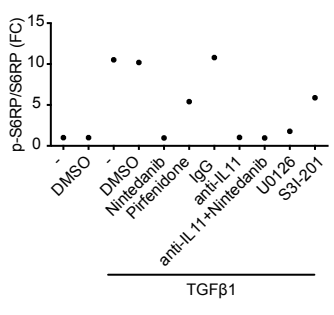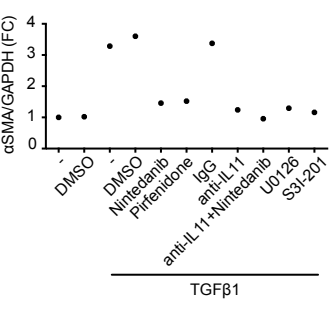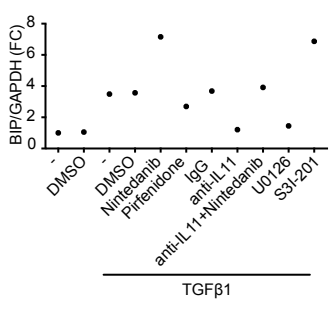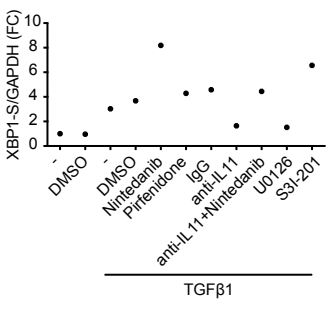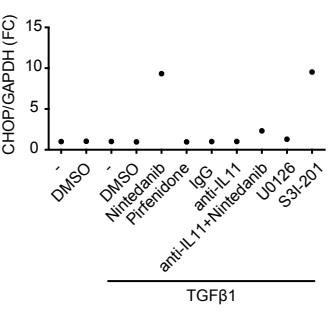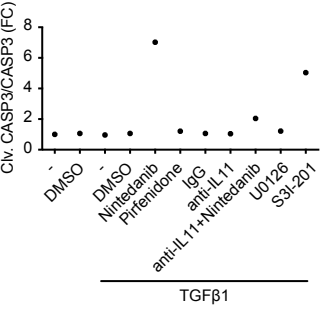

Supplement: Supplementary file 1 [file DataSheet2.PDF]
