# Supplementary material for: Molecular Dissection of Pro-Fibrotic IL11 Signaling in Cardiac and Pulmonary Fibroblasts
Source: Front Mol Biosci. 2021 Sep 28;8:740650. doi: 10.3389/fmolb.2021.740650 (PMC8505966; doi:10.3389/fmolb.2021.740650)
Supplement: Supplementary file 2 [file DataSheet4.docx]

**Supplementary Materials**


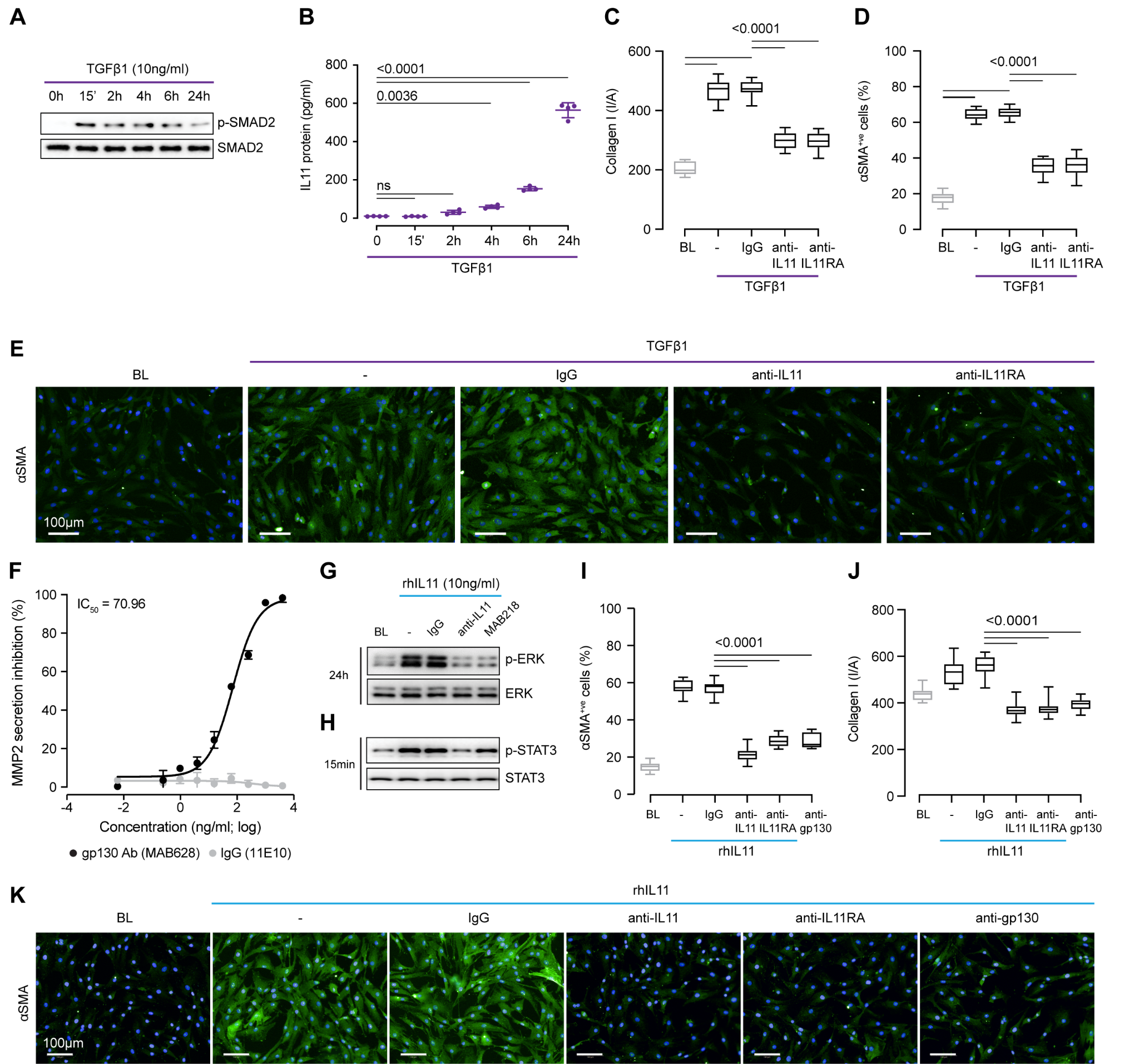


**Figure S1 TGFβ1 stimulates myofibroblast transformation in an IL11- and ERK-dependent manner.** (**A-B**) (A) SMAD2 activation status in the lysates (n=1) and (B) levels of IL11 protein in the supernatant from TGFβ1-stimulated HCFs over a time course (n=4). (**C-E**) Quantification of (C) Collagen I staining (n=14) and (D) ⍺SMA^+ve^ cells (n=14) and (E) representative IF images of ⍺SMA staining (n=3) in TGFβ1-stimulated HCFs in the presence of either IgG, anti-IL11, or anti-IL11RA. (**F**) Dose-response curve and IC_50_ value of IgG (11E10) and anti-human gp130 (MAB628, R&D Systems, range: 61 pg ml^-1^ to 4 µg ml^-1^; 4-fold dilution) in inhibiting MMP2 secretion in the supernatant from rhIL11-stimulated primary HCFs (n=2). (**G-H**) Western blot analysis of (G) ERK (24h) and (G) STAT3(15m) activation status in IL11-stimulated HCFs in the presence of IgG, anti-IL11, or a commercial neutralizing anti-IL11 (MAB218) (n=1).(**I-K**) Quantification of the effects of anti-IL11, anti-IL11RA, or anti-gp130 on (I) ⍺SMA (n=14) and (J) Collagen I (n=14) induction in IL11-stimulated HCFs and (K) their respective representative immunofluorescence images (n=3). (A-K) primary HCFs; 24h; IL11/TGFβ1 (10 ng/ml), IgG/anti-IL11/anti-IL11RA/MAB218/anti-gp130 (2 µg/ml), unless otherwise specified. (B, F) Data are shown as mean ± SD, (C-D, I-J) data are shown as box-and-whisker with median (middle line), 25th–75th percentiles (box) and min-max percentiles (whiskers); one-way ANOVA with Tukey’s correction. BL: baseline


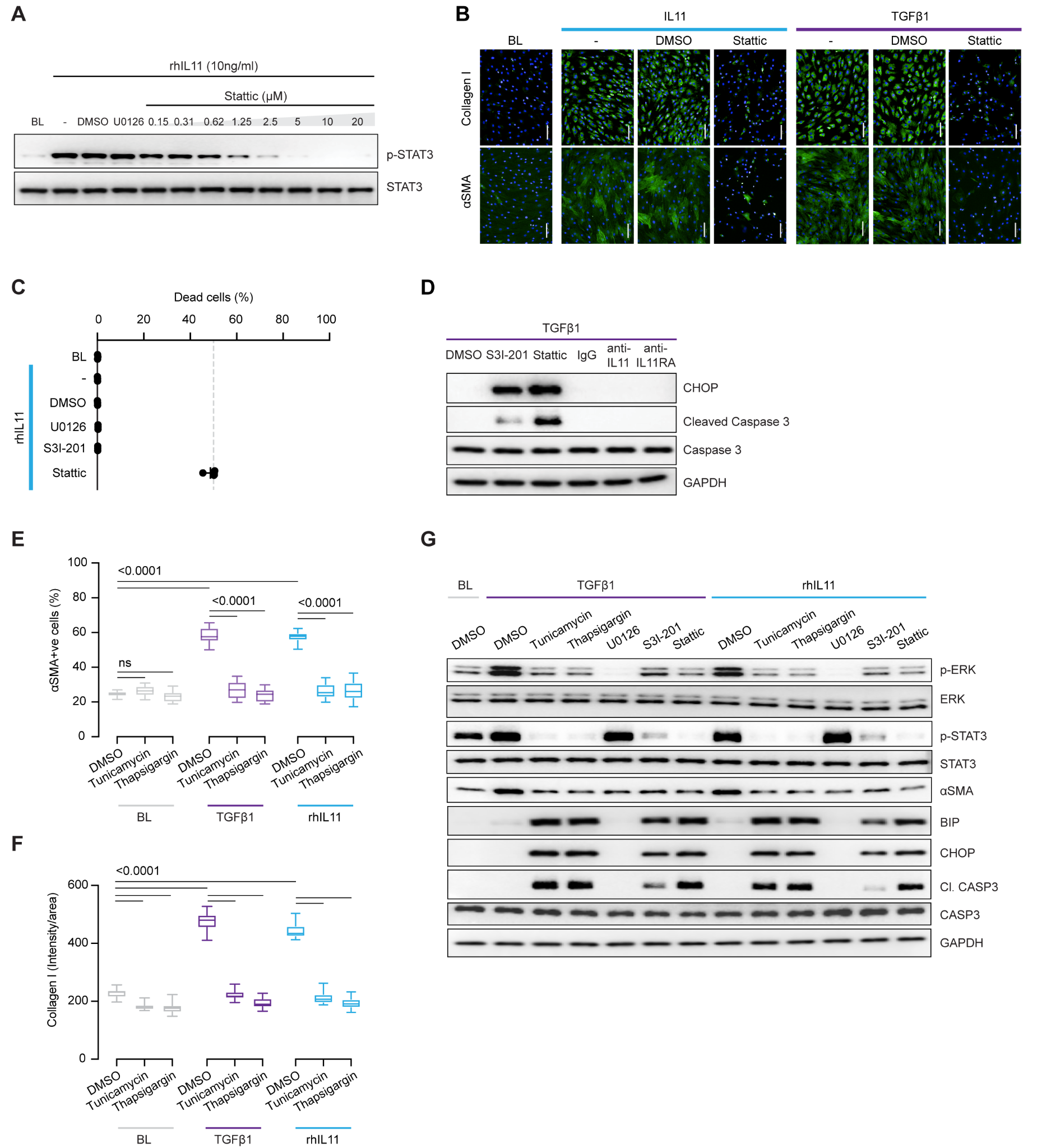


**Figure S2 STAT3 inhibition causes ER stress-related fibroblast dysfunction and cell death.** (**A**) Dose-dependent effects of increasing concentration of Stattic on STAT3 phosphorylation in IL11-stimulated HCFs at 15m time point (n=1). (**B**) Representative fluorescence images of ⍺SMA and Collagen I immunostaining in HCFs following stimulation with rhIL11 or TGFβ1 in the presence of Stattic (n=3) (**C**) Effects of U0126, S3I-201, or Stattic on cell viability as assayed by live/dead cell staining; data are shown as mean ± SD (n=3). (**D**) Comparison effects of S3I-201, Stattic, IgG, anti-IL11, and anti-IL11RA on Caspase3 activation and CHOP induction (n=1). (**E-F**) Quantification of (E) ⍺SMA^+ve^ cells and (F) Collagen I immunostaining from TGFβ1- or rhIL11- stimulated HCFs (n=14); data are shown as box-and-whisker with median (middle line), 25th–75th percentiles (box) and min-max percentiles (whiskers); one-way ANOVA with Tukey’s correction. (**G**) Western blots of pERK, ERK, pSTAT3, STAT3, ⍺SMA, BIP, CHOP, Cleaved Caspase3, Caspase3, and GAPDH from TGFβ1- or rhIL11-stimulated HCFs (n=1). (A-G) primary HCFs; 24h; IL11/TGFβ1 (10 ng/ml), U0126 (10 µM), S3I-201 (20 µM), Stattic (2.5 µM), IgG/anti-IL11/anti-IL11RA (2 µg/ml), Tunicamycin (5 µg/ml), Thapsigargin (300 nM) unless otherwise specified. BL: baseline


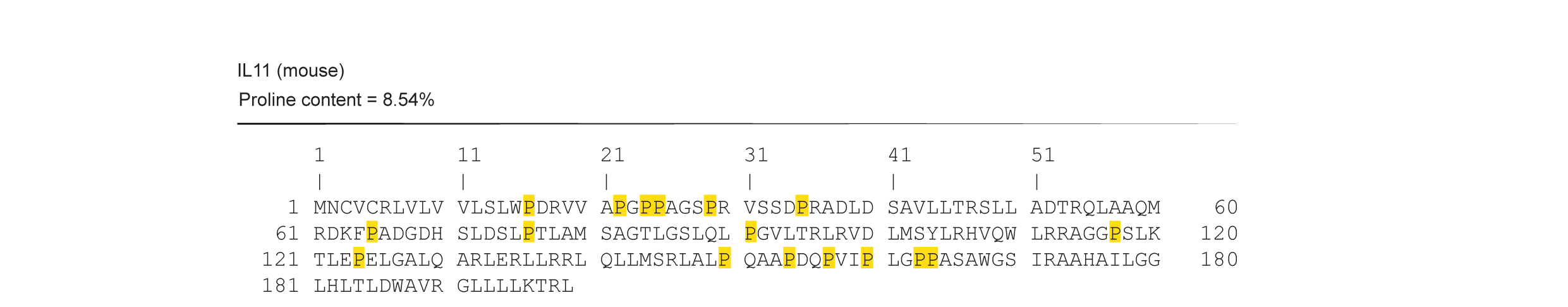


**Figure S3. Amino acid sequence of mouse IL11.** Proline residues highlighted in yellow.

**
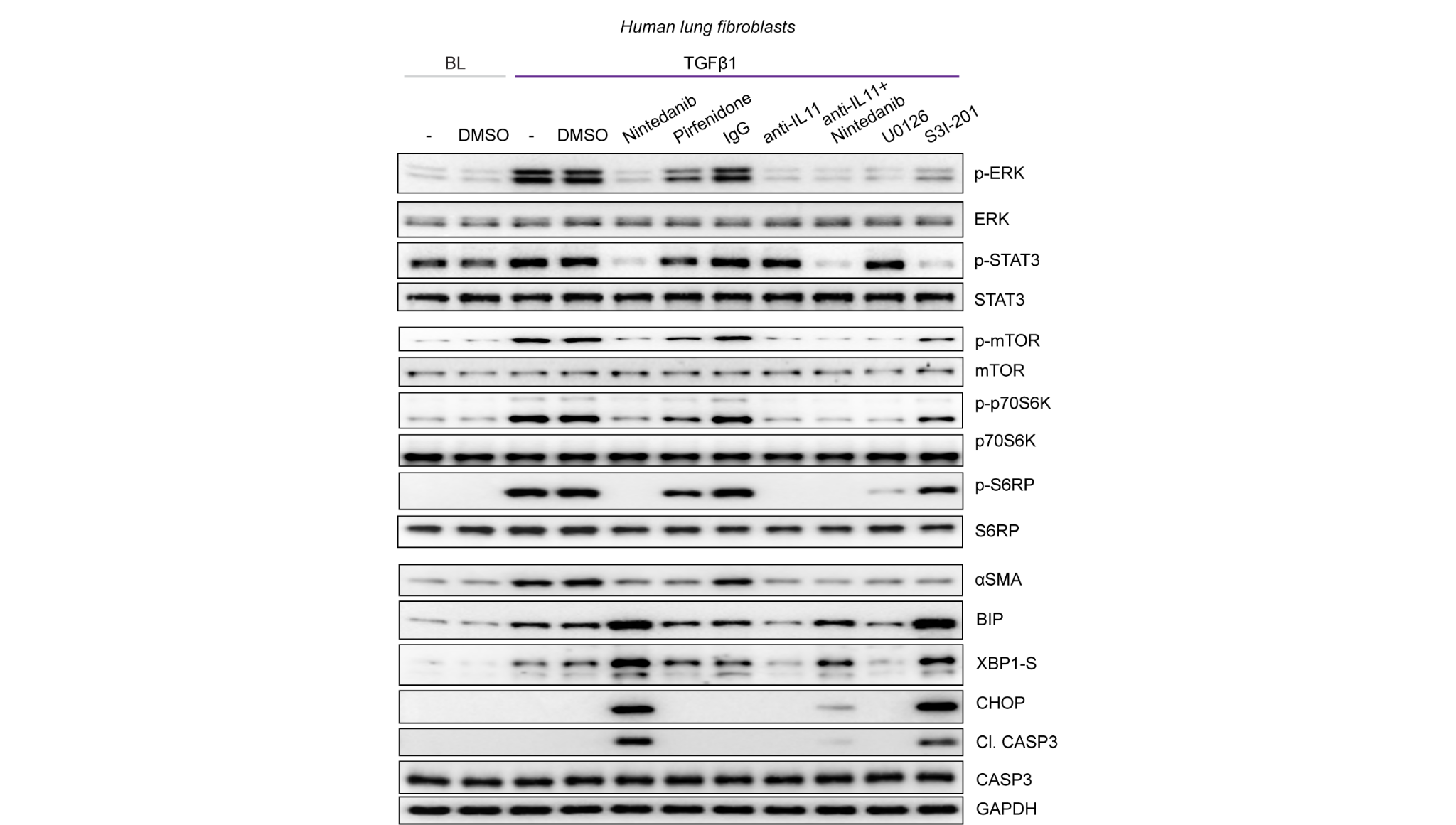
**

**Figure S4 Nintedanib, Pirfenidone and anti-IL11 have different anti-fibrotic mechanisms of action in lung fibroblasts.** Western blots showing activation status of ERK, STAT3, mTOR, p70S6K (T389), S6RP, and Caspase3, and protein expression of ⍺SMA, BIP, XBP1-S, and CHOP following treatment with nintedanib, pirfenidone, IgG, anti-IL11, a combination of nintedanib, anti-IL11, U0126 or S3I-201 from TGFβ1-stimulated human lung fibroblasts (24 hours, n=1); TGFβ1 (10 ng/ml), IgG/anti-IL11 (2 µg/ml), nintedanib (2 µM), pirfenidone (0.3 mg/ml), U0126 (10 µM), S3I-201 (20 µM), unless otherwise specified. BL: baseline
