# Supplementary material for: Molecular Dissection of Pro-Fibrotic IL11 Signaling in Cardiac and Pulmonary Fibroblasts
Source: Front Mol Biosci. 2021 Sep 28;8:740650. doi: 10.3389/fmolb.2021.740650 (PMC8505966; doi:10.3389/fmolb.2021.740650)

Uncropped western blot images

Figure 1A

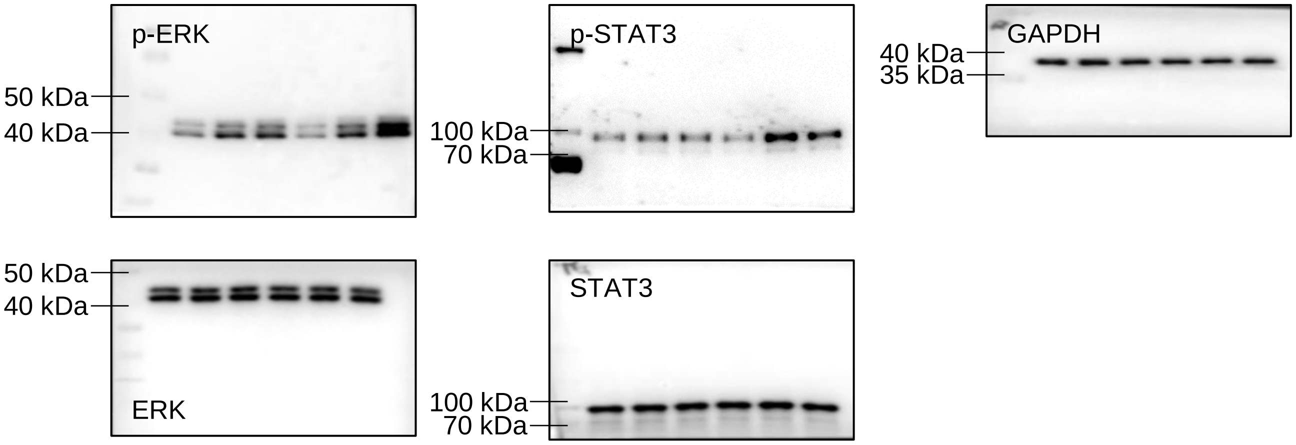

Figure 1C

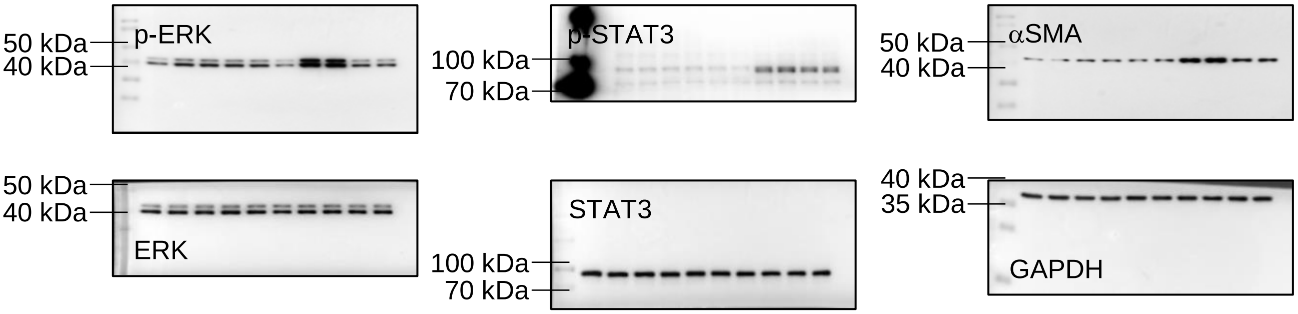

Figure 1E

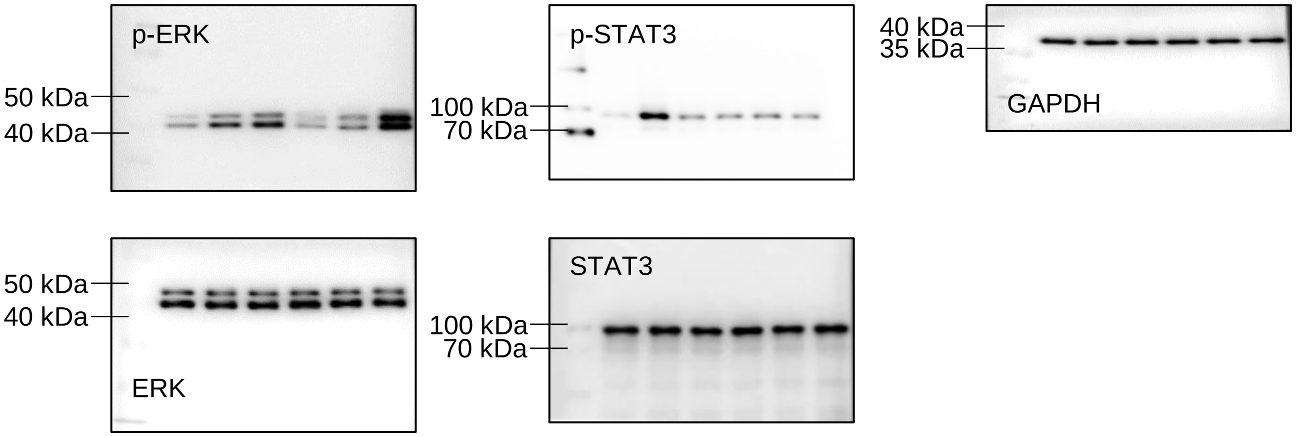

Figure 1F

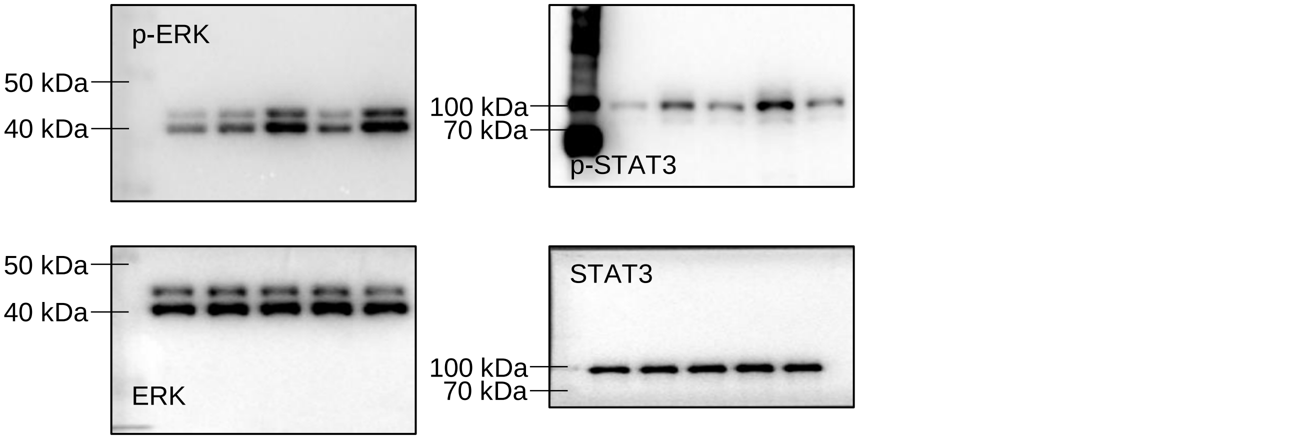

Figure 1G

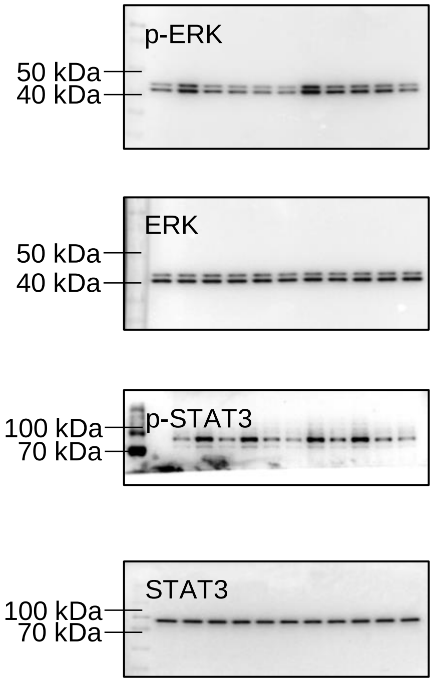

Figure 1H

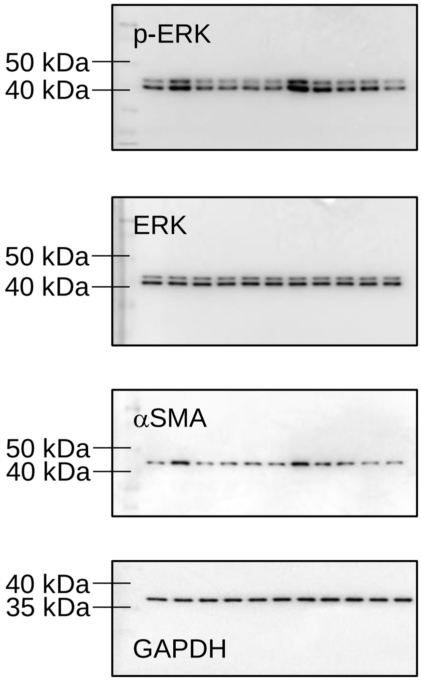

Figure 1K

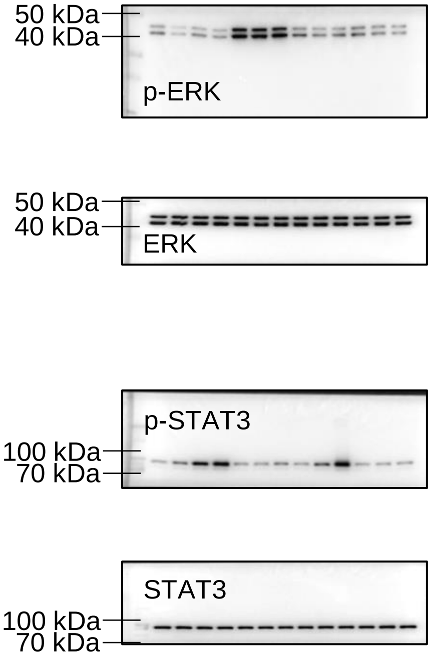

Figure 1L

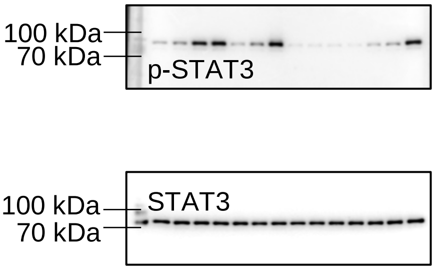

Figure 1M

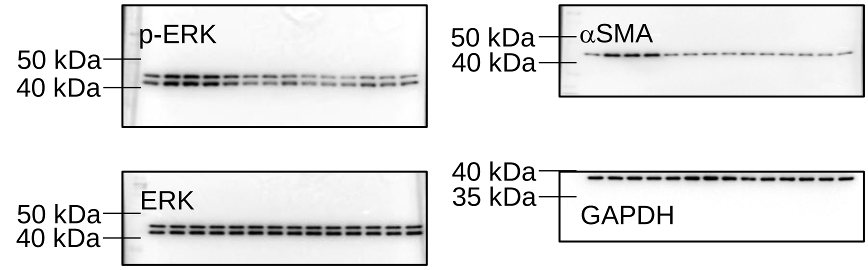

Figure 2E

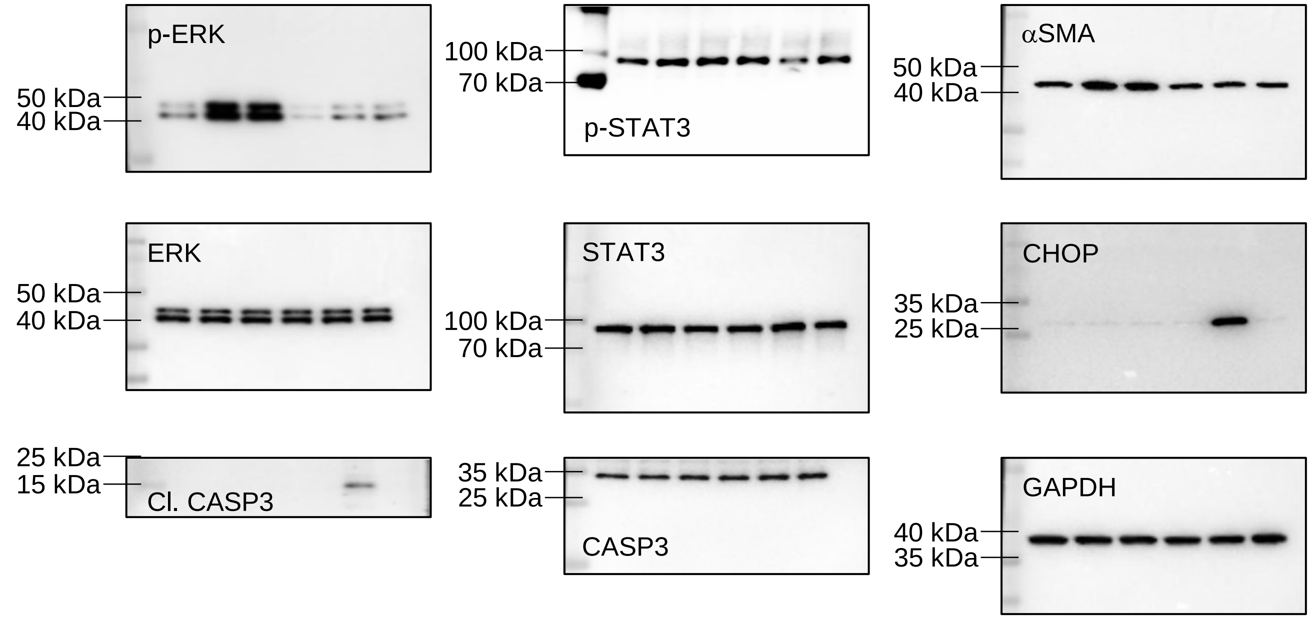

Figure 2J

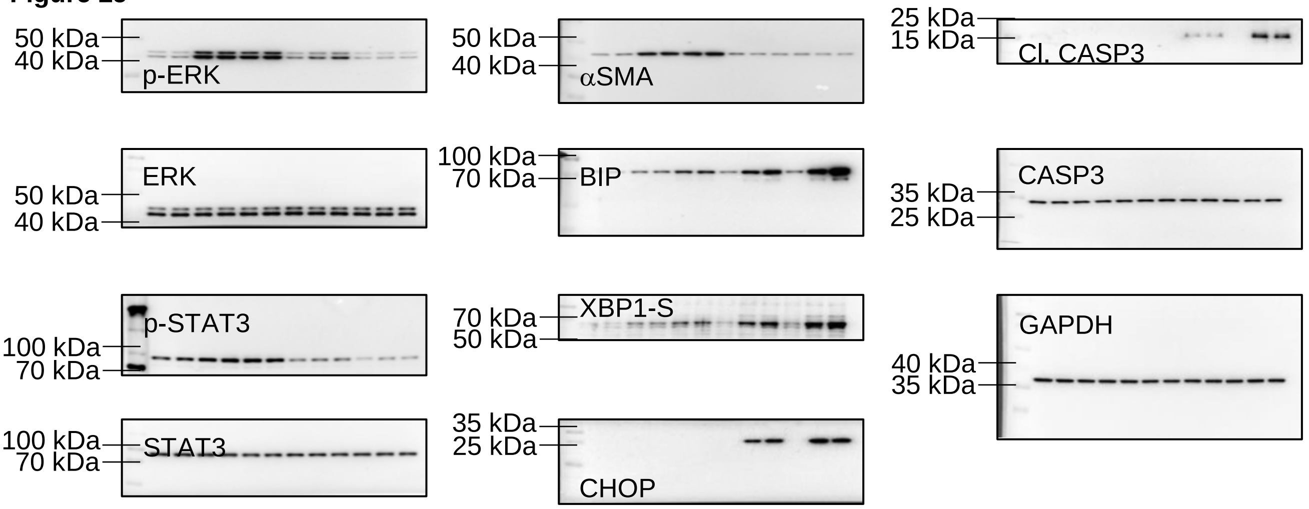

Figure 2K

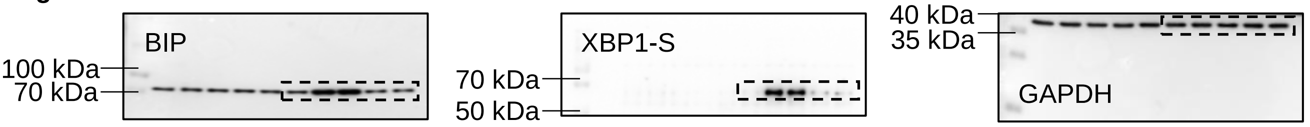

Figure 2M

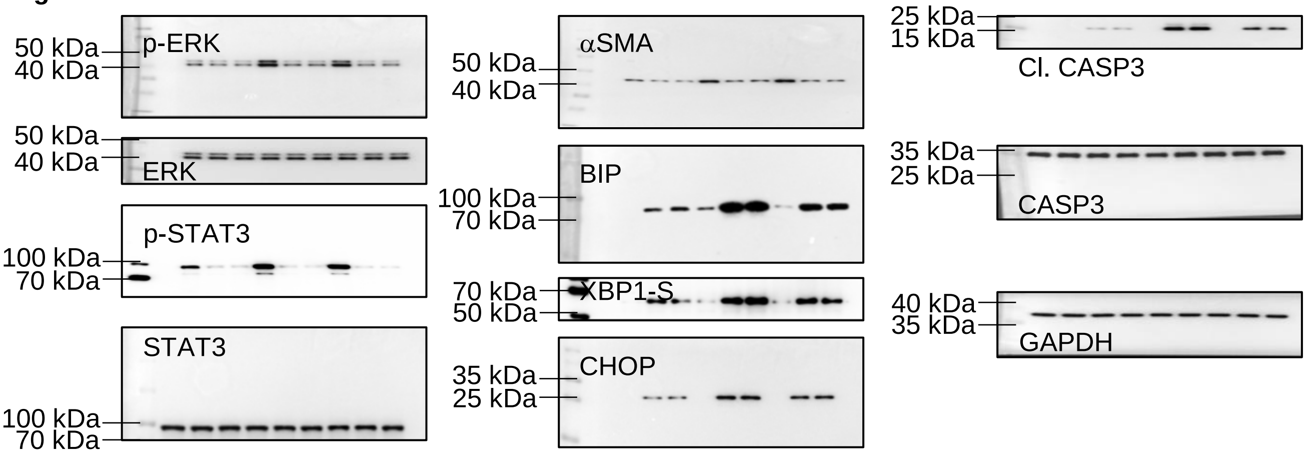

Figure 3E

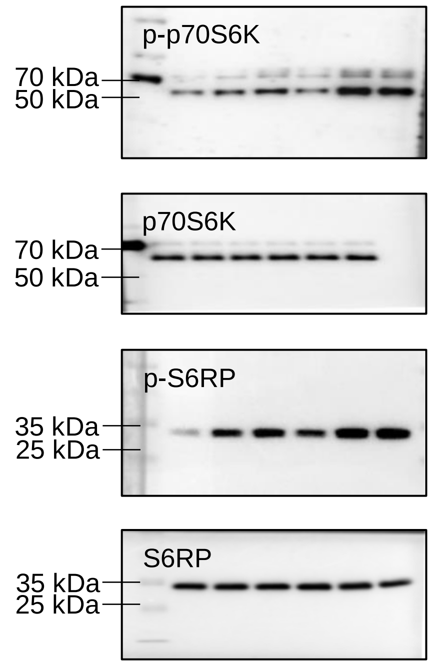

Figure 3F

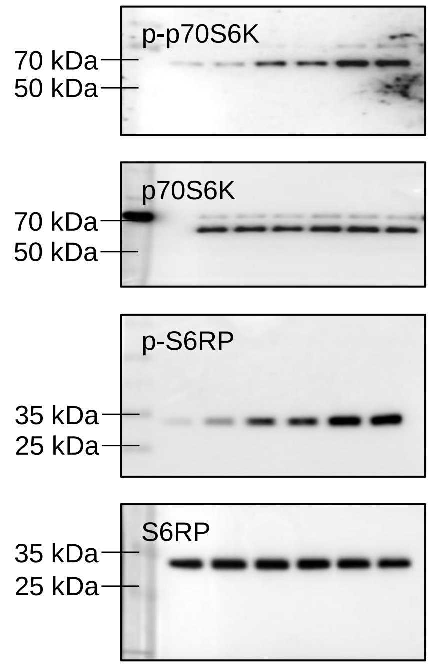

Figure 3G

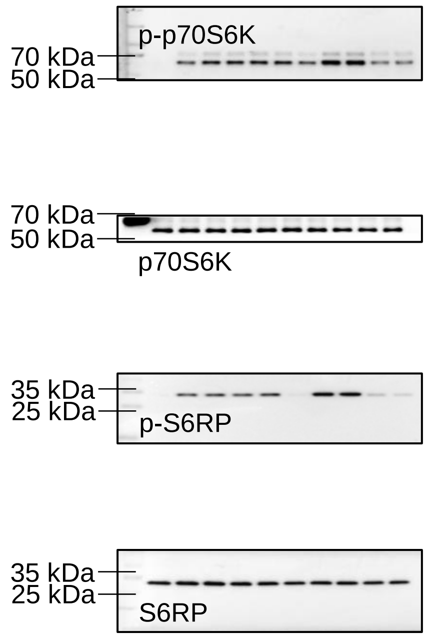

Figure 3H

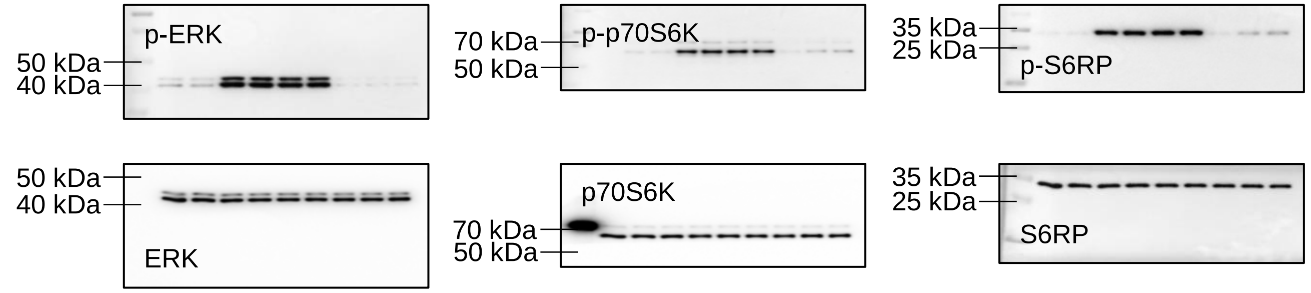

Figure 3I

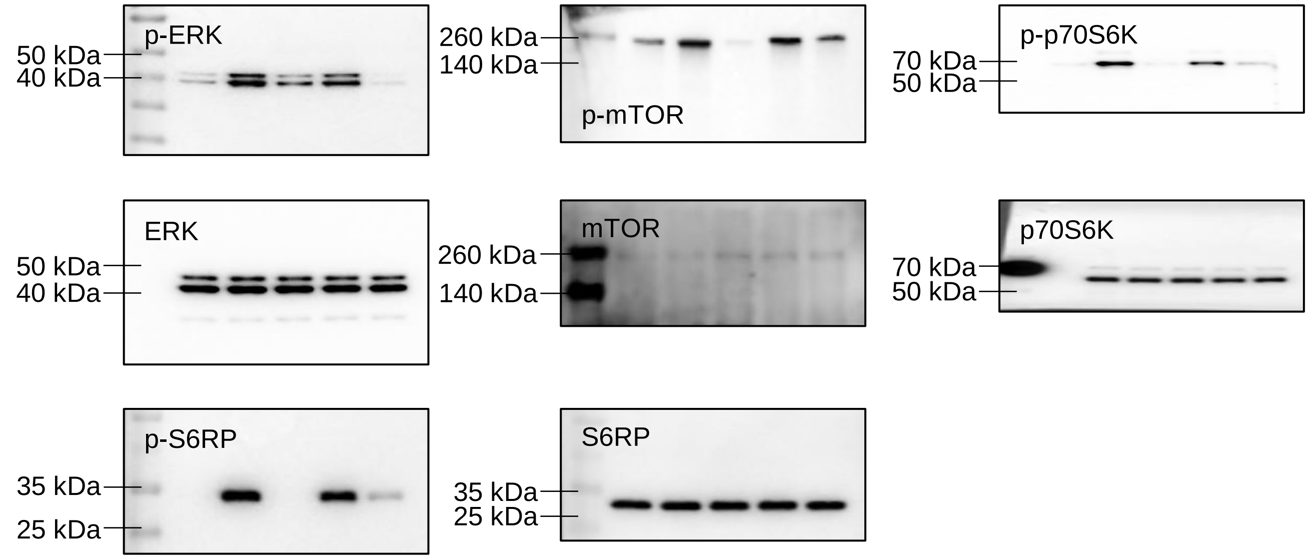

Figure 4F

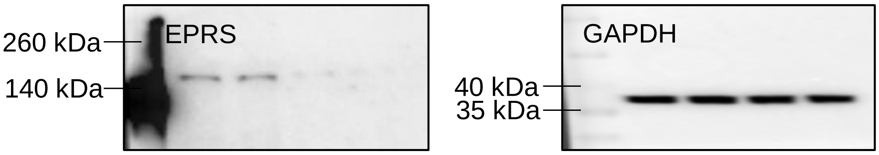

Figure 5A

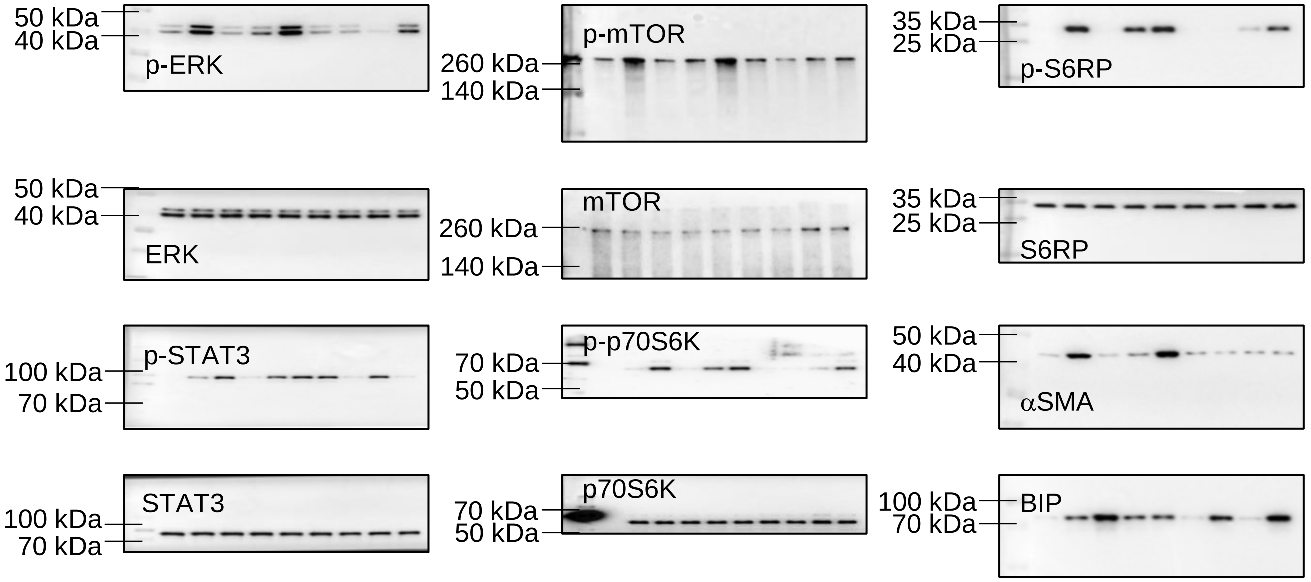

Figure 5A (continued)

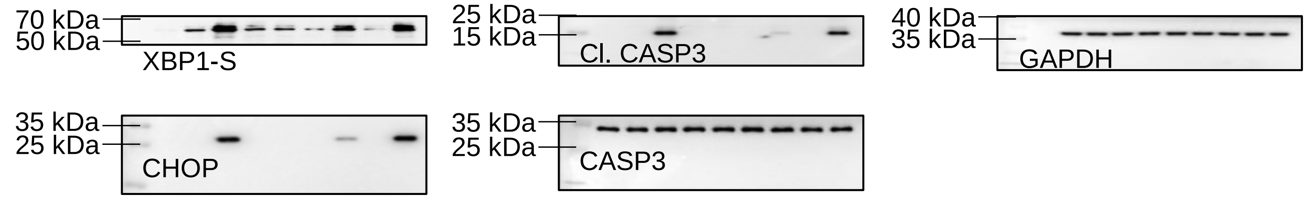

Figure 5B

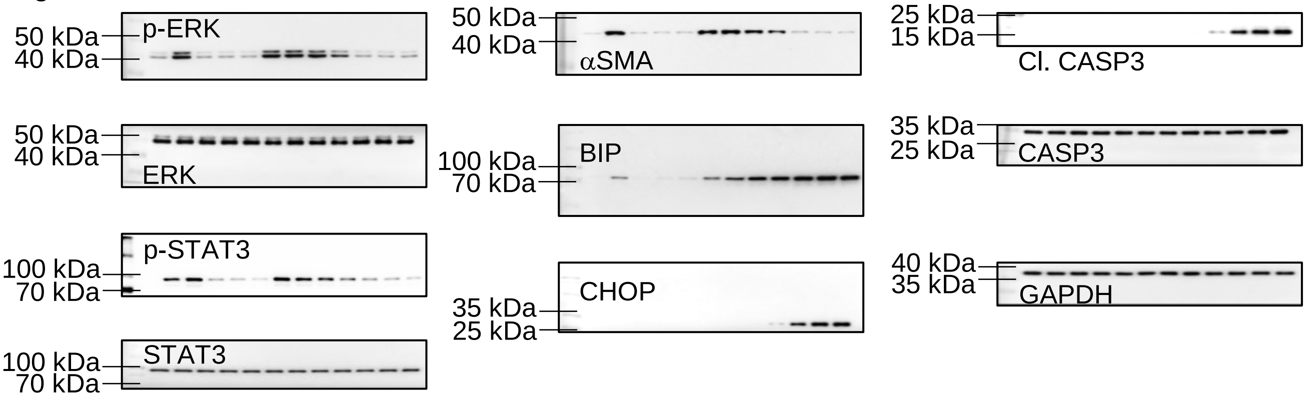

Figure 5C

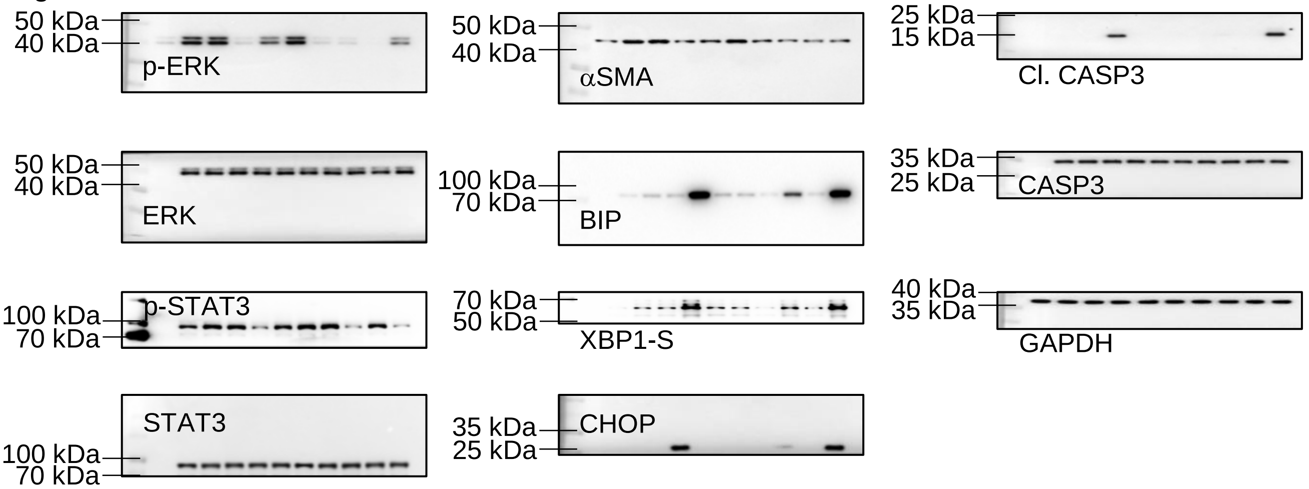

Figure 5D

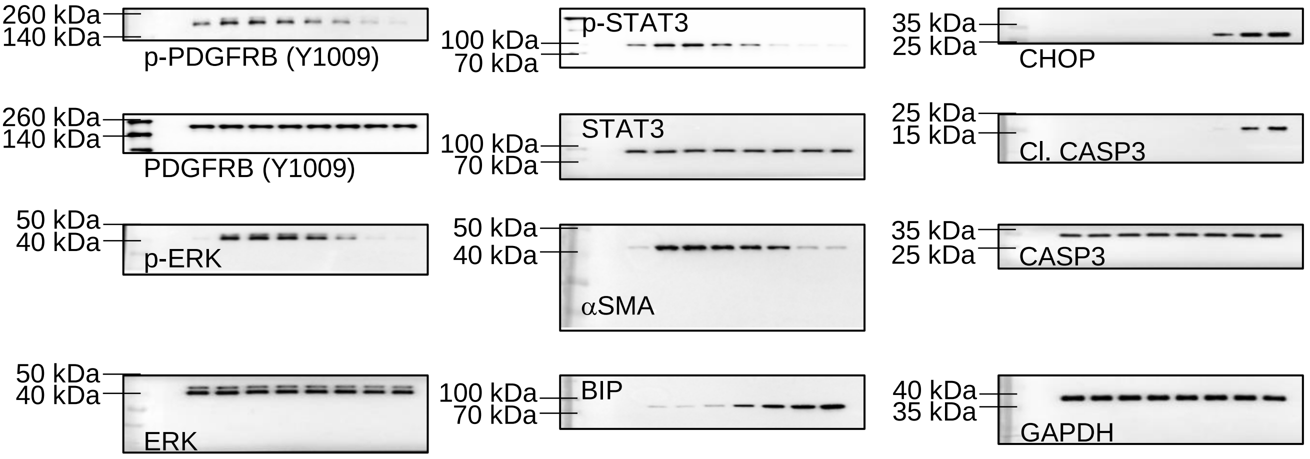

Figure S1A

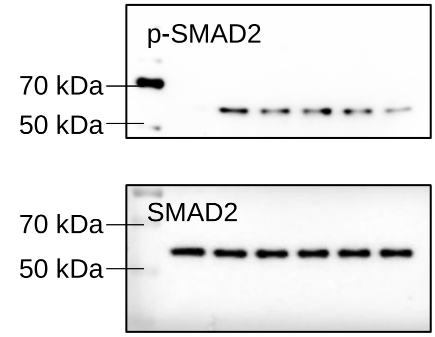

Figure S1G

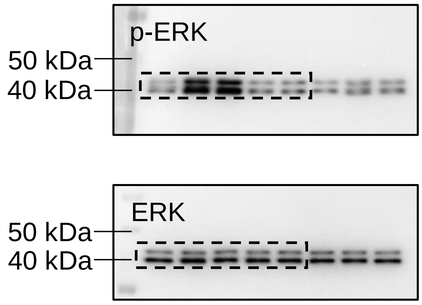

Figure S1H

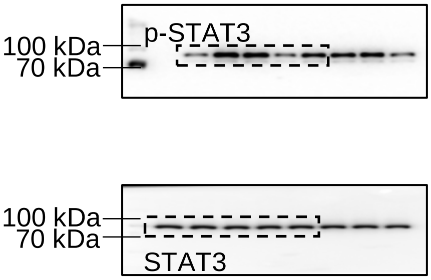

Figure S2A

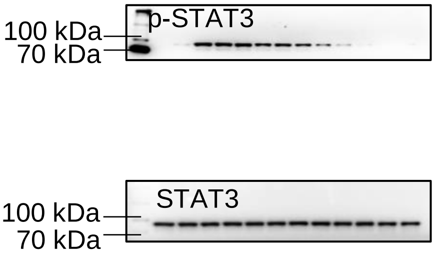

Figure S2D

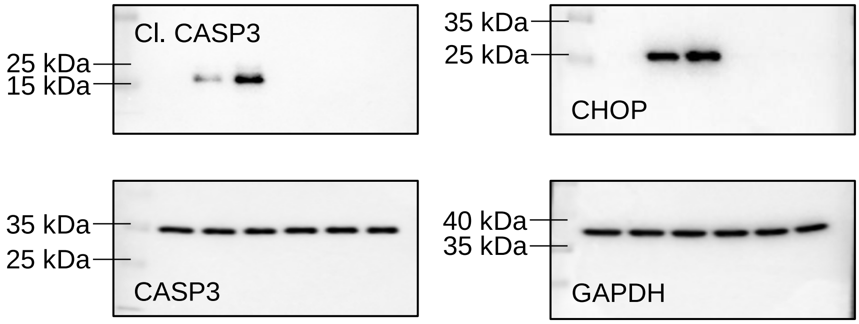

Figure S2G

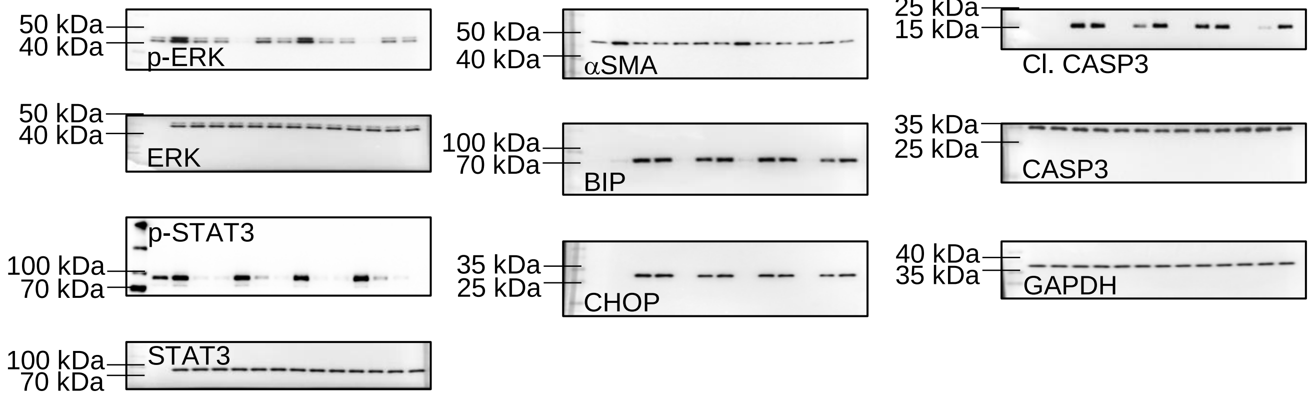

Figure S4

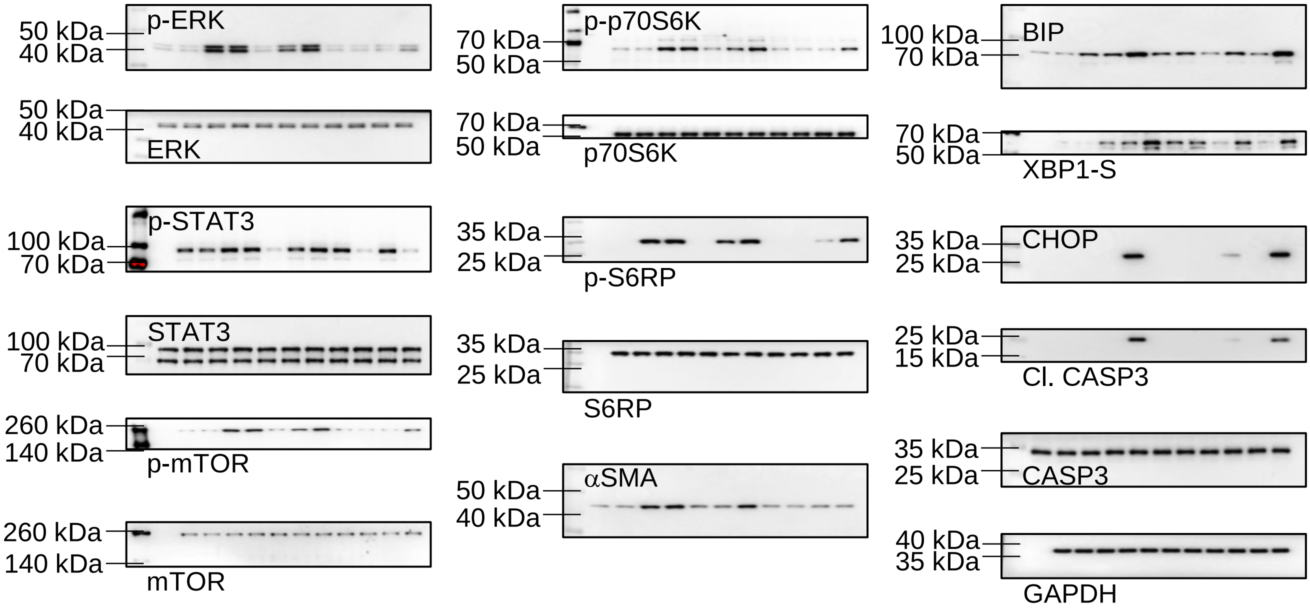

Supplement: Supplementary file 4 [file DataSheet1.PDF]
